# Effects of aerobic exercise on event-related potentials related to cognitive performance: a systematic review

**DOI:** 10.7717/peerj.13604

**Published:** 2022-07-11

**Authors:** Julia Gusatovic, Mathias Holsey Gramkow, Steen Gregers Hasselbalch, Kristian Steen Frederiksen

**Affiliations:** Danish Dementia Research Centre, Department of Neurology, Rigshospitalet, University of Copenhagen, Copenhagen, Denmark

**Keywords:** Exercise, Aerobic exercise, Event-related potentials, ERP, EEG, Cognition, Electroencephalography, Cognitive task, Evoked potential

## Abstract

**Introduction:**

Aerobic exercise interventions may affect different cognitive domains such as attention, working memory, inhibition, *etc*. However, the neural mechanisms underlying this relationship, remains uncertain.

**Objective:**

To perform a systematic review on exercise intervention studies that use event-related potentials (ERPs) as outcome for cognitive performance.

**Methods:**

We identified studies through searches in four databases reporting the effects of either an acute bout or chronic exercise on any ERP associated with cognitive performance. Study population included participants >17 years of age with or without a diagnosis.

**Results:**

A total of 5,797 records were initially identified through database searching of which 52 were eligible for inclusion. Most studies were of acute aerobic exercise with moderate intensity. Results were heterogenious across studies, but there was a trend that ERP amplitude increased and (to a lesser extent) latencies decreased post-exercise. The P3 ERP was the most often reported ERP.

**Conclusion:**

Heterogeneity across studies regarding methodology limited the possibility to draw definitive conclusions but the most consistent findings were that acute aerobic exercise was associated with higher amplitudes, and to a lesser extent shorter latencies, of ERPs.

## Introduction

Aerobic exercise has been shown to improve brain health as well as cognitive functioning (Barha et al., 2017; Laurin et al., 2001; Pope, Shue & Beck, 2003; Smith et al., 2010). The physiological links between aerobic exercise and cognitive function may be facilitated through many different mechanisms, *e.g.*, secretion of neuromodulators (such as brain-derived neurotrophic factor (BDNF)), neurogenesis, increased brain plasticity and increased brain blood flow (Waters et al., 2020), but so far evidence for the biological mechanisms underlying this relationship remain sparse. Moreover, it may be speculated that different underlying mechanisms may mediate the effects of acute exercise interventions, which may be immediate and short-lived, versus longer exercise interventions, which conversely may be slower to develop and more long-lasting. A number of studies have found an effect of long-term exercise on the hippocampus, white matter and the cortical mantle (Colcombe et al., 2006; Erickson et al., 2011; Pajonk et al., 2010). Although imaging studies of acute and immediate exercise effects are lacking, findings examining the effects of exercise on BDNF further indicate differential effects of acute and longer-term exercise. Specifically, BDNF was found to be elevated immediately following an acute bout of exercise, but not following 3 months of exercise (Krogh et al., 2014; Tsai et al., 2021). Nevertheless, both acute and chronic exercise seem to have beneficial effects on the brain. Acute aerobic exercise has been shown to facilitate learning mechanisms (Perini et al., 2016; Voss et al., 2021) and improve cognition in post-recovery period following exercise in healthy subjects (Erickson et al., 2019). A meta-analysis showed that acute bouts of aerobic exercise improved cognitive task performance (Lambourne & Tomporowski, 2010) and a large body of literature supports this notion (Ratey & Loehr, 2011). A systematic review concluded that aerobic exercise interventions exceeding one month are associated with modest improvements in attention and processing speed, executive functioning and memory (Smith et al., 2010). It could be theorized that chronic exercise enhances cognitive aspects by modulation of brain structure and vascular proliferation and perfusion, which develops over time, and acute exercise works by the immediate effects of neurosecretion related to exercise. However, differences in precisely which cognitive domains that are affected, and by which underlying mechanisms, by acute and chronic exercise respectively are yet to be investigated in more detail.

One method to quantify the impact of aerobic exercise on brain function and cognitive performance is by event-related potentials (ERPs), which has been widely used in studies investigating perception, attention and cognitive functioning (Helfrich & Knight, 2019). ERPs are small electrical potentials generated in the cortex (or subcortical generators) in response to a specific stimulus or event and can be measured noninvasively by electroencephalography (EEG) or magnetoencephalography (MEG) using scalp electrodes (Woodman, 2010). Information about neural activity such as early sensory perception processes and higher-level processing such as attention, cortical inhibition, response selection, error monitoring, memory update, and other cognitive functions (Duncan et al., 2009; Polich, 2007) can be obtained as different ERP components vary according to stimulus type and type of cognitive task. The most studied endogenous ERP is the P3 (300–500 ms post-stimulus), which is interpreted as an index of ability to sustain attention to a target. P3 is difficult to localize and most studies agree that P3 (P3b) has multiple dipole sources, *e.g.*, the hippocampus and the parahippocampal areas, the insula, the temporal lobe, the occipital cortex and the thalamus (Sokhadze et al., 2017). Another frequently studied ERP is the N2, associated with categorization, perceptual closure, inhibitory control and attention focusing (Sokhadze et al., 2017) and is generated by frontal and anterior cingulate cortex (Heil et al., 2000). Due to the noninvasiveness, ease of use and temporal resolution of the technique and the fact that it can be applied immediately after an intervention, ERPs represent an attractive method of capturing neural effects of acute bouts of exercise (Pedroso et al., 2017). ERPs are in general considered to express different components of executive functions, such as processing time and the amount of cognitive resources allocated to the perception and processing of an event/task. Especially P3 seems significantly impacted by exercise in most studies.

The objective of the present study was to carry out a systematic review of studies reporting on the effects of both acute and chronic exercise on ERPs related to cognitive performance and associated behavioral measures such as accuracy and reaction time. Furthermore, we aimed to investigate whether exercise intensity was correlated with changes in ERPs.

## Methods

### Study design and protocol registration

This study is a systematic review. Results were reported in accordance with the guidelines provided by the Preferred Reporting Items in Systematic Reviews (PRISMA) statement.

A protocol for the systematic review was registered on 09/11/2020 in the PROSPERO database (PROSPERO ID: 218808) (https://www.crd.york.ac.uk/prospero/).

### Participants, intervention, comparators

We included single group, parallel group and cross-over studies with both randomized and non-randomized allocation involving participants older than 17 years, with no history of epilepsy and sleep disorders. Interventions could be either acute aerobic exercise (single bout) or chronic aerobic exercise (>2-weeks of duration). Interventions were divided according to their exercise intensity, which for light-intensity exercise were set at <50% of HR _max_, moderate-intensity at 50%–80% of HR _max_ and high-intensity at >80% of HR _max_. If data was not available in the studies corresponding authors were contacted, and if not possible the exercise intensity level reported was adopted. The outcome of interest was difference in ERP parameters such as latency or amplitude obtained by either EEG or MEG pre- to post intervention or between intervention group and control group pre- to post intervention. Data on effects on behavioral measures (reaction time and accuracy) were also extracted. Any paradigm judged by the authors to elicit a cognitive performance response (based on the literature on the subject) was accepted. ERPs related to processing of emotional stimuli (regardless of whether there was a cognitive element) were not included. The stimuli evoking the ERPs trials could be in any sensory modality. No limits in terms of publication year were set. Only studies in the English language and full research articles were eligible for inclusion.

### Search strategy

Searches were performed in the following databases: PubMed, Web of Science, Cochrane Library and Embase. The databases were searched from inception to the 06/NOV/2020. Mesh-terms and keywords (from the literature and thesauruses, including “exercise”, “evoked potential”, “event-related potential”, “EEG”, “electroencephalography”, “MEG”, “magnetoencephalography”) were searched for in each database.

### Study selection, data extraction and data items

**Figure 1:**
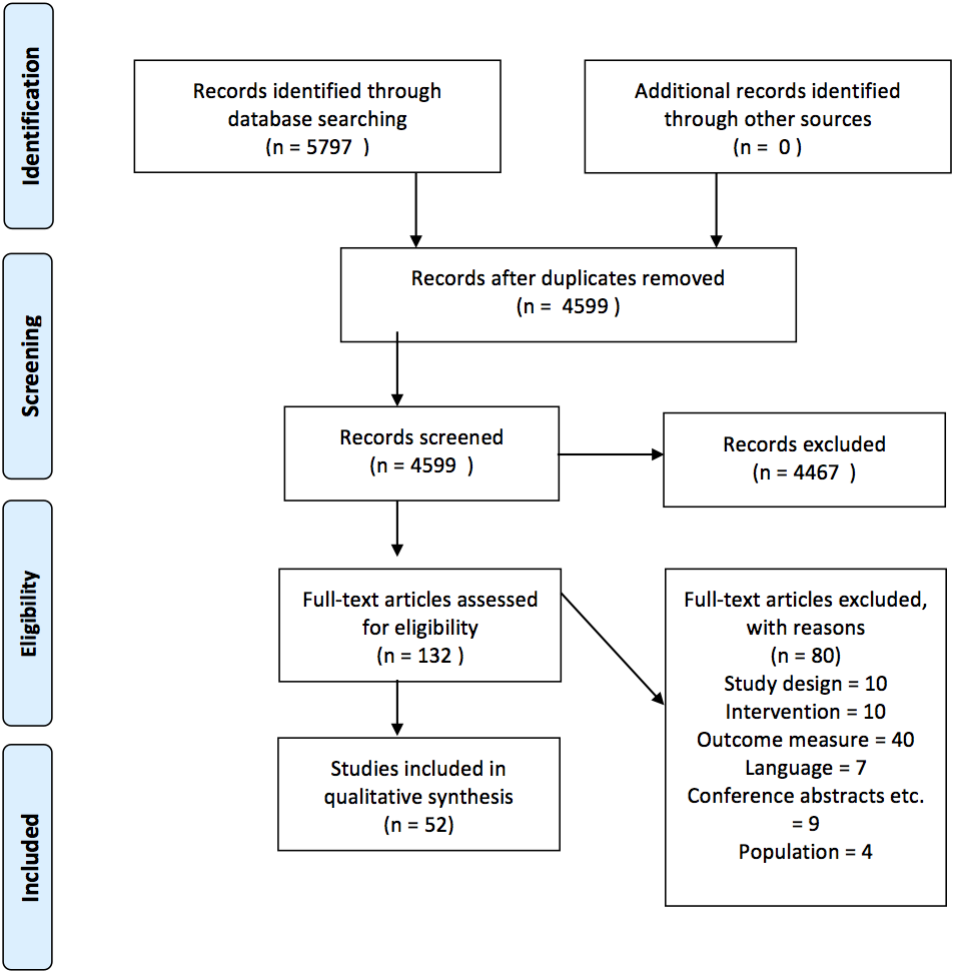
Prisma flow diagram.

Three authors screened and selected the included studies (JG, MG, KF). Authors were blinded with regards to the results of each authors’ screening results. The authors initially screened articles on title and abstract level. Subsequently, full text articles for those identified in the first step were retrieved and assessed for final inclusion. Any disagreement with regards to whether a study could be included was resolved by consensus (JG and KF) (see Fig. 1 for flow-chart and supplementary table for more detailed explanations on exclusion reasons). Relevant data was extracted by the same author (JG) and reviewed by another author (MG). Data was extracted in an Excel data extraction sheet that was piloted using four studies before being applied to the rest of the studies. The following items were extracted: number of participants, gender, age, diagnosis, study design, characteristics of comparator and intervention (type, duration, exercise composition, intensity and how it was measured), methods (EEG/MEG used, cognitive paradigm and sensory modality, ERP outcome measurements), reported effect of the intervention on ERP measurements and behavioral results. A risk-of-bias assessment was carried out using Cochrane’s Risk of Bias version 1.

### Synthesis of results

Due to large heterogeneity and according to the protocol, a qualitative synthesis of results was carried out.

## Results

### Included studies

The study selection process is outlined in Fig. 1. A total of 5,797 articles were identified through database searching. Fifty-two articles representing 52 unique studies (unit of analysis) were eligible for inclusion.

### Characteristics of included studies

The 52 studies identified comprised a total of 1,734 participants. Forty-one studies investigated acute exercise interventions with the following characteristics: sample size range was 7–72, the vast majority (37 studies) included participants with a mean age range of 18–40 years, and most (38 studies) included healthy subjects only. In terms of exercise intensity, 25 studies examined the effects of moderate-intensity exercise, five examined high-intensity exercise and seven studied a combination of low-, moderate- and high-intensity exercise. The intensity could not be established in four studies. Exercise durations were primarily single bouts lasting 9–40 min and most studies used either treadmill running or exercising on a stationary bicycle. In the control conditions participants were engaged in non-exercise related activities as resting or magazine reading.

Eleven studies investigated chronic exercise interventions comprising a total of 625 participants. Sample size range was 28–141. In five studies the mean age range was 18–40, and in five studies mean age was over 60 years. One study reported a participants’ age range of 40–60 years. Three studies were in healthy subjects and eight studies investigated different patient populations. Six studies investigated moderate intensity exercise, one study investigated low and high intensities and two studies examined moderate and high intensities. Two studies were of unknown exercise intensities. The duration and frequency of the exercise interventions ranged from 30–60 min sessions three times per week for three weeks to 30–60 min sessions three times per week for 24 weeks. Most studies used a combination of running/jogging and stationary bicycling. Control conditions were non-exercise related activities (*e.g.*, usual care). All studies used EEG for the ERP assessments. See Tables 1 and 2 for study characteristics for each study included.

**Table 1:** Study characteristics (acute aerobic exercise).

**References**	**Design**	**N**	**Gender (% male)**	**Age**	**Diagnosis**	**Comparator(s)**	**Intervention(s)**	**Comments**
Xie et al. (2020)	Counterbalanced within-subject	16	100	24.5 ± 5.09	Healthy	Sedentary	Ergometer cycling exercise test (single bout 30 min)	
Tsai et al. (2014)	Parallel group	60	100	EI(H): 22.20 ± 2.17. EI(L): 23.10 ± 2.20. NEI: 22.20 ± 1.70	Healthy	Rest and magazine reading	Motordriving treadmilling (single bout 33 min)	
Yagi et al. (1999)	Single group	24	50	20 ± 2	Healthy	Baseline	Ergometer cycling (single bout 10 min)	
Walsh et al. (2019)	Counter-balanced within-subject	25	36	22.4 ± 3.5	Healthy	Nature documentary watching and rest	Body-weight exercises (single bout 11 min)	Exercises included burpees, squat jumps and other aerobic components
Scudder et al. (2012)	Counter-balanced within-subject	37	51	19.7 ± 1.3	Healthy	Paper reading	Motor-driven treadmilling (single bout 30 min)	
Swatridge et al. (2017)	Counter-balanced within-subject	9	67	57.8 ± 11.4	Chronic stroke	Rest	Semirecumbent stepper (single bout 20 min)	
Chacko et al. (2020)	Counter-balanced within-subject	15	53	26.8 ± 5.1	Healthy	Internet browsing	Ergometer cycling (single bout 40 min)	
Akatsuka et al. (2015)	Counterbalanced within-subject	10	100	19.8 (SD not stated)	Healthy	Rest	Treadmill running (single bout 15 min)	
Kamijo et al. (2009)	Counter-balanced within-subject	24	100	Older: 65,5 ± 1,5. Younger: 21.8 ± 0.6	Healthy	Baseline	1. Light intensity ergometer cycling 2. Moderate intensity ergometer cycling (single bouts 25 min)	The participans were divided in two groups according to age (young vs old)
Kao, Wang & Hillman (2020)	Counter-balanced within-subject	23	48	19.2 ± 0.6	Healthy	Rest	Treadmilling (single bout 20 min)	
Aly & Kojima (2020)	Parallel group	40	70	CG: 23.10 ± 2.20. IG: 22.90 ± 2.40	Healthy	Inactive resting	Ergometer cycling (single bout 20 min)	
Tsai et al. (2018)	Parallel group	66	42	AE: 65.48 ± 7.53. RE: 66.05 ± 6.64. control: 64.50 ± 6.95	Mild cognitive impair-ment	1. Rest and magazine reading 2. Resistance exercise	Ergometer cycling (single bout 40 min)	
Wollseiffen et al. (2016)	Single group	11	45	36.5 ± 7	Healthy	Baseline	Running (6 h)	The participants were specifically trained and experienced in ultra-marathon running
Wen & Tsai (2020)	Parallel group	32	0	IG: 33,13 ± 6,27 CG: 32.92 ± 7.17	Healthy	Sitting quietly	combination of aerobic dancing and resistance training (single bout 40 min)	The study was performed in obese women
Chu et al. (2015)	Counter-balanced within-subject	21	90	21.50 ± 4.68	Healthy	Sedentary (reading)	Treadmilling (single bout 30 min)	
Shibasaki et al. (2019)	Single group	15	100	20.8 ± 0.9	Healthy	Baseline	interval cycle exercise on ergometer bicycle (four bouts of 15 min)	
Milankov et al. (2012)	Single group	10	0	22.4 average, range 19–24	Healthy	Baseline	Ergometer interval cycling (three bouts of 10 min)	
Ligeza et al. (2018)	Counter-balanced within-subject	18	100	24.9 ± 2.2	Healthy	Sitting and reading sports-related magazines	1. Ergometer cycling moderate intensity continous 2. Ergometer cycling interval high intensity (single bouts 24 min)	
Pontifex et al. (2015)	Counter-balanced within-subject	34	47	19.3 ± 0.9	Healthy	Restful sitting	Treadmilling (single bout 20 min)	
Kamijo et al. (2007)	Counter-balanced within-subject	12	100	25,7 ± 0,7	Healthy	Baseline	1. Ergometer cycling mild intensity 2. Ergometer cycling moderate intensity 3. Ergometer cycling hard intensity (22 min single bouts)	
Wang et al. (2020)	Parallel group	60	100	exercise: 32.73 ± 7.15 control: 32.40 ± 7.76	Heroin addiction	Resting and reading about heroin addiction treatments	Stationary cycle exercise (single bout 30 min)	
Chu et al. (2017)	Counter-balanced within-subject	20	90	20.42 ± 1.16	Healthy	Reading	Treadmilling (single bout 30 min)	
Zhou & Qin (2019)	Parallel group	72	50	20.07 ± 0.15	Healthy	Resting and reading	Cycling pedaling (single bout 25 min)	
Rietz et al. (2019)	Counter-balanced within-subject	26	100	21.5 ± 2.52	Healthy	Sitting and nature documentary watching	Ergometer cycling (single bout 30 min)	
Themanson & Hillman (2006)	Counter-balanced within-subject	28	50	higher fit: 20.1 ± 1.7. Lower fit: 20.6 ± 2.4	Healthy	Resting and reading	Treadmilling (single bout 30 min)	
Dimitrova et al. (2017)	Parallel group	56	54	younger: 23.2 ± 2.7. Older: 70.7 ± 5.4	Healthy	Baseline	1. Cybercycle riding (normal exercise) 2. Cybercycle riding (exergaming)	
Kao et al. (2017)	Counter-balanced within-subject	64	42	19.2 ± 0.8	Healthy	Seated rest	1. Continous aerobic exercise treadmilling (single bout 20 min) 2. High-intensity interval training (single bout 9 min)	
Won et al. (2017)	Counter-balanced within-subject	12	100	24.8 ± 2	Healthy	Seated rest	1. Treadmilling (single bout 20 min) 2. Futsal (single bout 30 min)	
Bae & Masaki (2019)	Counter-balanced within-subject	29	48	21.4 ± 1.2	Healthy	Quiet resting	Treadmilling (single bout 30 min)	
Hwang et al. (2019)	Single group	30	0	20.4 range 18–22	Healthy	Baseline	Treadmilling (single bout 20 min)	
Magnié et al. (2000)	Single group	20	100	High fit group: 21.2. Low fit group: 22.9. No SD	Healthy	Baseline	Exercise protocol on a bicycle (until volitional exhaustion was reached)	
Drapsin et al. (2012)	Single group	24	0	Judo players: 20.61 ± 3.09. Healthy: 21.06 ± 4.09	Healthy	Baseline	1. Ergometer cycling 60% HR max 2. ergometer cycling 75% HR max 3. ergometer cycling 90% HR max (single bouts 10 min)	
Kao et al. (2018)	Counter-balanced within-subject	36	50	21.5 ± 3.1	Healthy	Seated rest	1. Treadmilling high intensity 2. Treadmilling moderate intensity (single bouts 20 min)	
Wu et al. (2019)	Counter-balanced within-subject	30	57	21.17 ± 1.32	Healthy	1. resistance exercise 2. reading	Cycle ergometry (single bout 30 min)	
Jain et al. (2014)	Counter-balanced within-subject	12	100	between 18 and 21	Healthy	Seated rest	Treadmilling (single bout, terminated on achieving any of three criteria)	(i) Volitional exhaustion, (ii) HR within 10 bpm of age predicted maximum or (iii) Rating of perceived exertion of ≥17 onBorg’s Scale
Winneke et al. (2019)	Counter-balanced within-subject	11	36	25.64 ± 3.78	Healthy	Rest	Stationary bicycling (single bout 20 min)	
Nakamura et al. (1999)	Single group	7	100	34.6 ± 4.7	Healthy	Baseline	Jogging (single bout 30 min)	
Yagi et al. (1998)	Single group	10	50	mean 20.6 (no SD)	Healthy	Baseline	Ergometer cycling (single bout 10 min)	
Takuro, Nishihira & Soung-Ryol (2009)	Counter-balanced within-subject	14	100	24.2 ± 1.3	Healthy	NR	Stationary cycling (single bout 30 min)	
Chang et al. (2017)	Counter-balanced within-subject	30	57	22.67 ± 1.52	Healthy	Sedentary reading	Ergometer cycling (single bout 30 min)	
Chang et al. (2015)	Parallel group	30	53	EG: 21.67 ± 3.77. CG: 20.17 ± 1.53	Healthy	Sedentary reading	Spinning wheel exercise	The participants were highly fit amateur basketball players

**Notes.**

Shows characteristics of studies using acute aerobic exercise interventions.

EI(H) exercise intervention (high-intensity) EI(L) exercise intervention (low-intensity) NEI No exercise intervention CG control group IG intervention group NR not registered

**Table 2:** Study characteristics (chronic aerobic exercise).

**References**	**Design**	**N**	**Gender (% male)**	**Age**	**Diagnosis**	**Comparator(s)**	**Intervention(s)**	**Comments**
Wang et al. (2017)	Parallel group	50	88	IG: 32.3 ± 6.97 CG: 34.76 ± 7.96	Metham-phetamine dependency	Usual care	Aerobic exercise i.e., Cycling, jogging, or jump rope (30 min × 3/week in 12 weeks)	
Pedroso et al. (2018)	Parallel group	50	FE: 40 SG: 31 HC: 36	FE and SG group: 78.0 ± 5.6 CG: 74.6 ± 5,3	Alzheimers disease	1. healthy control group 2. Social gathering (AD patients)	Exercises that stimulates aerobic endurance, flexibility, muscular resistance, and balance (60 min × 3/week in 12 weeks)	
Tsai et al. (2017)	Parallel group	64	100	O-ex: 66.88 ± 4.74. C-ex: 66.15 ± 4.90. Control: 65.70 ± 3.54	Healthy	1. balance and stretching (control) 2. Table tennis (open-skill exercise)	Bikeriding or brisk walking/jogging (closed-skill) (40 min × 3/week in twenty-four weeks)	
Olson et al. (2017)	Parallel group	30	20	21.1 ± 2.0	Major depressive disorder	Light-intensity stretching	Treadmilling og ergometer cycling (45 min × 3/week in eight weeks)	
Overath et al. (2014)	Single group	28	17	43.3 ± 9.7	Migraine	Baseline	Aerobic endurance programme: walking or interval jogging (40 min × 3/week in ten weeks)	
Chen et al. (2020)	Parallel group	44	Missing	Control: 33.87 ± 1.98. High-intensity: 32.73 ± 1.31. Moderate- intensity: 29.40 ± 1.19	Metham-phetamine-dependency	Normal daily routine	1. Ergometer cycling (moderate-intensity) 2. Ergometer cycling (hard-intensity) (40 min × 3/week in twelve weeks)	
Zhao et al. (2020)	Parallel group	64	100	29.38 ± 0.56	Metham-phetamine-dependency	Usual care	1. Cycling on stationary bike (moderate intensity) 2. Cycling on stationary bike (high intensity) (40 min × 3/week in twelve weeks)	
Özkaya et al. (2005)	Parallel group	44	68	CG: 72.3 ± 2.1. ST: 75.8 ± 2,8. ET: 70.9 ± 3.1	Healthy	1. No exercise 2. Strength training	Running track (50 min × 3/week in nine weeks)	
Brush et al. (2022)	Parallel group	55	32	20.23 ± 2.39	Major depressive disorder	Stretching	Treadmill and ergometer cycling (45 min × 3/week in eight weeks)	
Gajewski & Falkenstein (2018)	Parallel group	141	40	70.9 ± 5.2	Healthy	1. Cognitive training 2. No-contact control group 3. Social control group	Cardiovascular, aerobic, and strength exercises (90 min × 2/week in sixteen weeks)	
Tsai et al. (2019)	Parallel group	55	31	AE: 66.00 ± 7.68. RE: 65.44 ± 6.76. Control: 65.17 ± 7.00.	Mild cognitive impairment	1. Resistance exercise 2. Static stretching exercise	Ergometer cycling and treadmilling (40 min × 3/week in sixteen weeks)	

**Notes.**

The table shows characteristics of studies using chronic aerobic exercise interventions.

IG intervention group CG control group FE functional exercise group SG social gathering HC healthy control O-ex open-skill exercise group C-ex closed-skill exercise group ST strength training ET endurance training AE aerobic exercise group RE resistance exercise group

### Acute exercise intervention results

**Figure 2:**
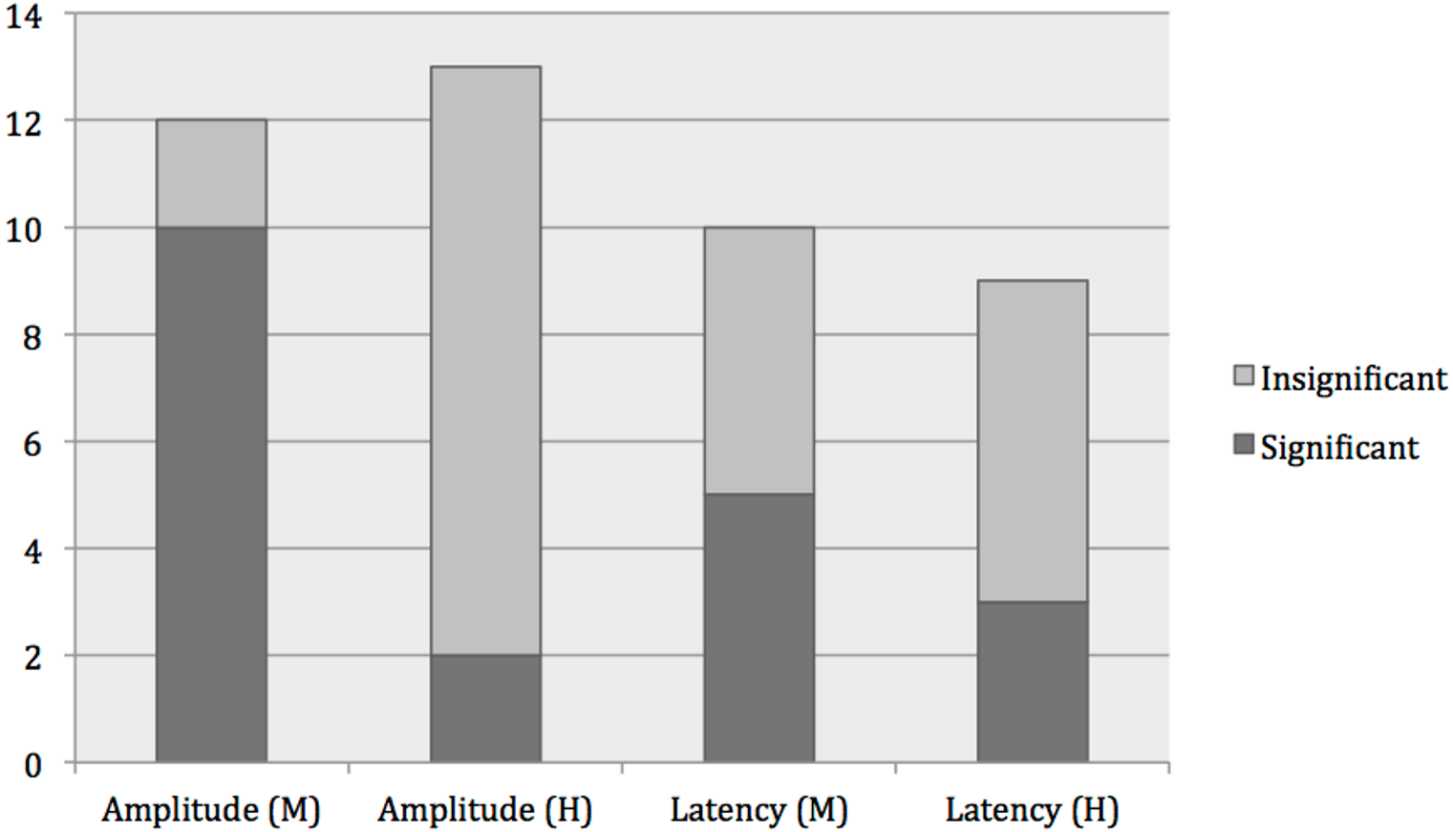
Effects of moderate-intensity exercise vs. high-intensity exercise on ERPs. Results from studies using flanker task and acute aerobic exercise interventions, with either moderate-intensity (M) or high-intensity (H) interventions. Significant results included (1) increased amplitude after intervention in all studies, except for one study that showed a significantly decreased amplitude after intervention, and (2) decreased latencies in all studies after intervention.

Due to the large heterogeneity across studies in terms of the paradigms used, ERPs investigated and methods employed, general trends in the data are difficult to extract. A total of 21 different ERPs were examined and seventeen different cognitive paradigms to elicit ERPs were used with flanker task as the most often used paradigm (12 studies). The most frequently reported ERP was the P3 (33 studies) followed by N2 and N140. Regarding P3, each study sometimes reported results for P3 amplitudes and latencies from more than one experiment as several paradigms were used to elicit the P3 or several exercise intensities were investigated, and thus numbers of experiments are reported in the following. A total of 27 experiments out of 41 experiments reported significant effects of the exercise intervention on amplitude (25 increased, two decreased) and 16 reported effects on latency (two increased, 14 decreased). Fifteen of the 27 experiments reported significant effects on both amplitude and behavioral results (mainly decreased reaction time, only one study reported effect on accuracy (increased)) and seven experiments out of the 16 experiments reported a significant effect on latency and behavioral results (decreased reaction time). Looking across exercise intensities, there was a tendency that effects on amplitudes were significant mainly in interventions using moderate intensity exercise across all ERPs and paradigms used (see Fig. 2). Twenty-six out of 31 studies that investigated moderate-intensity interventions reported significant increases in one or more ERP component post-exercise. Conversely, out of the eleven studies that did not report any effect on ERP amplitude, two studies were of moderate intensity exercise. See Table 3 for all the results of studies using acute aerobic exercise.

**Table 3:** Acute aerobic exercise results.

**References**	**Diagnosis**	**Recorded time after exercise**	**Outcome measures**	**Paradigm**	**ERP results**	**Behavioural results**
**Light-intensity exercise results**						
Kamijo et al. (2009)	Healthy	2 min	P3	Flanker task (visual)	Amplitude: P3↔	Accuracy:↔
					Latency: P3↓	Reaction time:↔
Kamijo et al. (2007)	Healthy	3 min	P3	Flanker task(visual)	Amplitude: P3↑	Accuracy:↔
					Latency: P3↓	Reaction time:↓
**Moderate-intensity exercise results**						
Tsai et al. (2014)	Healthy	15-20 min	P3 and CNV	Visuospatial attention task(visual)	Amplitude: P3↑ CNV↑	Accuracy:↔
					Latency: P3↔ CNV↔	Reaction time:↓
Yagi et al. (1999)	Healthy	Within 10 min	P3	Oddball task (visual and auditory)	Amplitude: P3↔	Accuracy:↔
					Latency: P3↔	Reaction time:↔
Scudder et al. (2012)	Healthy	20,2 ± 6,4min	P3 and N2	AX-continuous performance tasks (visual)	Amplitude: P3↑ N2↔	Accuracy:↑
					Latency: P3↔ N2↔	Reaction time:↔
Akatsuka et al. (2015)	Healthy	5 min	N140	Go-/No-go task (somato-sensory)	Amplitude: N140↑	Accuracy:↔
					Latency: N140↔	Reaction time:↔
Kamijo et al. (2009)	Healthy	2 min	P3	Flanker task (visual)	Amplitude: P3↑	Accuracy:↔
					Latency: P3↓	Reaction time:↓
Kao, Wang & Hillman (2020)	Healthy	30 min	P3	Serial N-back task (visual)	Amplitude: P3↑	Accuracy:↔
					Latency: P3↔	Reaction time:↔
Aly & Kojima (2020)	Healthy	HR returned to within 10% of pre-ex	P2, N2c and P3	Flanker task (visual)	Amplitude: P2↑ P3↑ N2c↑	Accuracy:↔
					Latency: P2↔ P3↔ N2c↔	Reaction time:↓
Wen & Tsai (2020)	Healthy	HR returned to within 10% of pre-ex	P2, N2 and P3	Stroop task (visual)	Amplitude: N2↓ P3↑ P2↔	Accuracy:↔
					Latency: N2↓ P3↓ P2↔	Reaction time:↔
Chu et al. (2015)	Healthy	Within 10 min	P3 and N1	Stop-signal task (visual)	Amplitude: P3↑ N1↔	Accuracy: NR
					Latency: P3↑ N1↔	Reaction time:↓
Shibasaki et al. (2019)	Healthy	Right after	N140 and P300	Go-/No-go task (somatosensory)	Amplitude: N140↓ P3↔	Accuracy:↔
					Latency: N140↔ P3↓	Reaction time:↔
Ligeza et al. (2018)	Healthy	HR returned to within 10% of pre-ex	N2 and P2b	Flanker task (visual)	Amplitude: P3↔ N2↑	Accuracy:↑
					Latency: NR	Reaction time:↓
Pontifex et al. (2015)	Healthy	HR returned to within 10% of pre-ex	P3a and P3b	Oddball task (visual)	Amplitude: P3a↔ P3b↑	Accuracy:↔
					Latency: P3a↔ P3b↔	Reaction time:↔
Kamijo et al. (2007)	Healthy	3 min	P3	Flanker task (visual)	Amplitude: P3↑	Accuracy:↔
					Latency: P3↓	Reaction time:↓
Chu et al. (2017)	Healthy	NR	P3 and conflict SP	Stroop color-word task (visual)	Amplitude: P3↑ SP↔	Accuracy:↔
					Latency: P3↔ SP↔	Reaction time:↓
Zhou & Qin (2019)	Healthy	HR returned to within 10% of pre-ex	P2, N2, P3b and N450	Stroop color-naming task (visual)	Amplitude: P2↑ N2↔ P3b↔ N450↔	Accuracy:↔
					Latency: P2↔ N2↔ P3b↔ N450↔	Reaction time:↔
Dimitrova et al. (2017)	Healthy	Within 20 min	NR	Stroop task (visual)	Amplitude: ERP↑	Accuracy: n↔
					Latency: NR	Reaction time:↓
Won et al. (2017)	Healthy	HR returned to within 10% of pre-ex	P3	Stroop color-word conflict task (visual)	Amplitude: P3↑	Accuracy: NR
					Latency: NR	Reaction time:↓
Hwang et al. (2019)	Healthy	90 min	N2	Facial Go-/No-go task	Amplitude: N2↓	Accuracy:↔
					Latency:↔	Reaction time:↔
Kao et al. (2018)	Healthy	NR	P3	Flanker task (visual)	Amplitude: P3↑	Accuracy:↔
					Latency: P3↔	Reaction time:↓
Wu et al. (2019)	Healthy	30 min	P3b and N1	Task-switching test	Amplitude: P3↑ N1↔	Accuracy:↔
					Latency: NR	Reaction time:↓
Winneke et al. (2019)	Healthy	2,56 min (range 2 –3,10)	N2 and P3	Flanker task	Amplitude: N2↑ P3↔	Accuracy:↔
					Latency: N2↔ P3↓	Reaction time:↔
Takuro, Nishihira & Soung-Ryol (2009)	Healthy	Right after + when HR had returned to pre-ex values	P3, early and late CNV	Go-/No-go reaction time task	Amplitude: P3↑ early CNV↑ late CNV↑ (only right after)	Accuracy: NR
					Latency: P3↔ early CNV↔ late CNV↔	Reaction time: NR
Chacko et al. (2020)	Healthy	Right after	BP, pN, N1, pN1, pP1 and P3	Discriminative response task	Amplitude: N1↓ all other↔	Accuracy:↔
					Latency: pN1↓ all other↔	Reaction time:↔
Milankov et al. (2012)	Healthy	NR	P3	Oddball task (auditory)	Amplitude: P3↔	Accuracy: NR
					Latency: P3↑	Reaction time: NR
Bae & Masaki (2019)	Healthy	HR returned to within 10% of pre-ex	P3	Task-switching paradigm (visual)	Amplitude: P3↑	Accuracy:↔
					Latency: P3↓	Reaction time:↓
Drapsin et al. (2012)	Healthy	Right after	P3	Oddball task (auditory)	Amplitude: P3↑	Accuracy:↔
					Latency: P3↔	Reaction time:↔
Swatridge et al. (2017)	Chronic stroke	Both 0, 20 and 40 min after	P3	Flanker task (visual)	Amplitude: P3↑ (40 min post-ex)	Accuracy:↔
					Latency: P3↓ (20 min post-ex)	Reaction time:↔
Tsai et al. (2018)	Mild cognitive impair-ment	HR returned to within 10% of pre-ex	P3	Flanker task (visual)	Amplitude: P3↑	Accuracy:↔
					Latency: P3↔	Reaction time:↓
Wang et al. (2020)	Heroin addiction	HR returned to within 10% of pre-ex	N2 and N2d	Go-/No-go task (visual)	Amplitude: N2↑ N2d↑	Accuracy:↑
					Latency: N2↔ N2d↔	Reaction time:↔
Chang et al. (2017)	Healthy	15 min after	N1, N2, P3 and N450	Stroop task (visual)	Amplitude: P3↑ N450↑ N1↔ N2↔	Accuracy:↔
					Latency: N450↓	Reaction time:↓
Chang et al. (2015)	Healthy	Within in 10 minutes	P3	Attention network task	Amplitude: P3↑	Accuracy:↔
					Latency: P3↔	Reaction time:↑
**High-intensity exercise results**						
Xie et al. (2020)	Healthy	Within 15 min	P3 and LPP	Flanker task (visual)	Amplitude: P3↔ LPP↑	Accuracy:↔
					Latency: NR	Reaction time:↓
Walsh et al. (2019)	Healthy	10 min	RewP	Novel gambling task (visual)	Amplitude: RewP↓	Accuracy:↔
					Latency: NR	Reaction time:↔
Ligeza et al. (2018)	Healthy	HR returned to within 10% of pre-ex	N2 and P2b	Flanker task (visual)	Amplitude: N2↔ P2b↔	Accuracy:↔
					Latency: NR	Reaction time:↔
Kamijo et al. (2007)	Healthy	3 min	P3	Flanker task (visual)	Amplitude: P3↔	Accuracy:↔
					Latency: P3↓	Reaction time:↓
Rietz et al. (2019)	Healthy	30 min	P3, CNV, N2	Continous performance task (visual)	Amplitude: P3↑ CNV↔ N2↔	Accuracy:↔
					Latency: P3↔ CNV↔ N2↔	Reaction time:↔
Rietz et al. (2019)	Healthy	41 min	P3, CNV, N2	Flanker task (visual)	Amplitude: P3↔ CNV↔ N2↔	Accuracy:↔
					Latency: P3↔ CNV↔ N2↔	Reaction time:↔
Rietz et al. (2019)	Healthy	54 min	P3, CNV, N2	Four-choice reaction-time task (visual)	Amplitude: P3↔ CNV↔ N2↔	Accuracy:↔
					Latency: P3↔ CNV↔ N2↔	Reaction time:↔
Kao et al. (2017)	Healthy	20 min	P3	Flanker task (visual)	Amplitude: P3↓	Accuracy:↔
					Latency: P3↓	Reaction time:↓
Kao et al. (2018)	Healthy	NR	P3	Flanker task (visual)	Amplitude: P3↔	Accuracy:↔
					Latency: P3↓	Reaction time:↓
Milankov et al. (2012)	Healthy	NR	P3	Oddball task (auditory)	Amplitude: P3↓	Accuracy: NR
					Latency: P3↔	Reaction time: NR
Themanson & Hillman (2006)	Healthy	HR returned to within 10% of pre-ex	Error negativity, error positivity and N2	Flanker task (visual)	Amplitude: all↔	Accuracy:↔
					Latency: all↔	Reaction time:↔
Drapsin et al. (2012)	Healthy	Right after	P3	Oddball task (auditory)	Amplitude: P300↔	Accuracy:↔
					Latency: P300↔	Reaction time:↔
Jain et al. (2014)	Healthy	HR returned to within 10% of pre-ex	P3	Oddball task (auditory)	Amplitude: P3↑	Accuracy: NR
					Latency: P3↓	Reaction time: NR
**Unknown intensity results**						
Wollseiffen et al. (2016)	Healthy	NR	P2 and N1	Chalkboard challenge	Amplitude: P2↔ N1↔	Accuracy:↔
					Latency: P2↔ N1↔	Reaction time:↔
Magnié et al. (2000)	Healthy	When body temperature and HR had returned to pre-exercise levels	P3, P2, N1 and N2	Oddball task (auditive)	Amplitude: P3↑ P2↔ N1↔ N2↔	Accuracy:↔
					Latency: P3↓ P2↔ N1↔ N2↔	Reaction time:↔
Nakamura et al. (1999)	Healthy	10 min	P3, P2, N100 and N2	Oddball task (auditory)	Amplitude: P3↑ P2↑ N100↔ N2↔	Accuracy:↔
					Latency: P3↔ P2↔ N100↔ N2↔	Reaction time:↔
Yagi et al. (1998)	Healthy	Right after	P3	Oddball task (visual)	Amplitude: P3↔	Accuracy:↔
					Latency: P3↔	Reaction time:↔

**Notes.**

Shows results for all ERPs investigated with different cognitive paradigms using acute aerobic exercise interventions. Arrows (↑) indicate increase in measure following exercise, arrows (↓) indicate decrease in measure following exercise and arrows (↔) indicate no difference.

HR Heart rate NR not registered CNV contingent negative variation LPP late positive potential RewP reward positivity

### Chronic exercise results

As with the reporting of acute exercise results, results for chronic exercise studies were very heterogeneous. Nine different paradigms were used, and eight different ERPs were measured. The most frequently recorded ERPs were P3 and N2 (each recorded in six studies). Regarding P3, three studies with moderate-intensity exercise reported effect on amplitude (all increased) and concomitant effects on behavior, but none reported effect on latency. The remaining three studies reported on unknown, moderate and moderate to low-intensity exercise interventions and showed no significant effect on P3 amplitude. For N2, four studies reported effect on amplitude (all increased) and one study reported an effect on latency (decreased). All studies that reported effects on ERP amplitude also reported an effect on behavioral results. Across intensity, as for acute exercise interventions, only studies of moderate-intensity exercise reported effects on ERPs. See Table 4 for all the results of studies using chronic aerobic exercise.

**Table 4:** Chronic aerobic exercise results.

**References**	**Exercise intensity**	**Population**	**Duration**	**ERPs reported**	**Paradigm**	**ERP results**	**Behavioural results**
Tsai et al. (2017)	M	Healthy	40 min × 3 /week in 24 weeks	P3	Task switching (visual)	Amplitude: P3↑	Accuracy:↔
						Latency: P3↔	Reaction time:↓
Tsai et al. (2017)	M	Healthy	40 min × 3 /week in 24 weeks	P3	N-back task (visual)	Amplitude: P3↑	Accuracy:↑
						Latency: P3↔	Reaction time:↔
Özkaya et al. (2005)	M	Healthy	50 min × 3 /week in 9 weeks	N1, P2, N2, and P3	Oddball task (auditory)	Amplitude: all↔	Accuracy: NR
						Latency: N2↓ P2↓ N1↔ P3↔	Reaction time: NR
Gajewski & Falkenstein (2018)	NR	Healthy	90 min × 2 /week in 16 weeks	P3a and P3b	N-back task (visual)	Amplitude: P3a↔, P3b↔	Accuracy:↔
						Latency: NR	Reaction time:↔
Wang et al. (2017)	M	Metham-phetamine dependency	30 min × 3 /week in 12 weeks	N2	Standard Go-/No-go task (visual)	Amplitude: N2↑	Accuracy:↑
						Latency: NR	Reaction time:↔
Wang et al. (2017)	M	Metham-phetamine dependency	30 min × 3 /week in 12 weeks	N2	Methamphetamine-related Go-/No-go task (visual)	Amplitude: N2↑	Accuracy:↑
						Latency: NR	Reaction time:↔
Pedroso et al. (2018)	L to M	Alzheimers disease	60 min × 3 /week in 12 weeks	P3	Oddball task (auditory)	Amplitude: P3↔	Accuracy: NR
						Latency: P3↔	Reaction time: NR
Olson et al. (2017)	M	Major depressive disorder	45 min × 3/week in 8 weeks	N2	Flanker task (visual)	Amplitude: N2↑	Accuracy:↔
						Latency: NR	Reaction time:↓
Overath et al. (2014)	NR	Migraine	40 min × 3/week in ten weeks	CNV	Trail making test	Amplitude: CNV↓	Accuracy:↔
						Latency: NR	Reaction time:↓
Overath et al. (2014)	NR	Migraine	40 min × 3/week in ten weeks	CNV	d2-letter cancellation test	Amplitude: CNV↓	Accuracy:↔
						Latency: NR	Reaction time:↓
Chen et al. (2020)	M	Metham-phetamine dependency	40 min/week in 12 weeks	N1 and P2	2-back task (visual)	Amplitude: N1↔ P2↔	Accuracy:↔
						Latency: NR	Reaction time:↔
Chen et al. (2020)	H	Metham-phetamine dependency	40 min × 3/week in 12 weeks	N1 and P2	2-back task (visual)	Amplitude: N1↓ P2↔	Accuracy:↑
						Latency: NR	Reaction time:↓
Zhao et al. (2020)	M	Metham-phetaminee dependency	40 min × 3/week in 12 weeks	N2 and P2	Temporal discounting task (visual)	Amplitude: P2↑ N2↑	Accuracy: NR
						Latency: NR	Reaction time:↓
Zhao et al. (2020)	H	Methamphetamine dependency	40 min × 3/week in 12 weeks	N2 and P2	Temporal discounting task (visual)	Amplitude: P2↔ N2↔	Accuracy: NR
						Latency: NR	Reaction time:↔
Brush et al. (2022)	M	Major depressive disorder	45 min × 3/week in 8 weeks	ERN	Flanker task (visual)	Amplitude: ERN↔	Accuracy: NR
						Latency: NR	Reaction time: NR
Brush et al. (2022)	M	Major depressive disorder	45 min × 3/week in 8 weeks	RewP	Doors task (visual)	Amplitude: RewP↔	Accuracy: NR
						Latency: NR	Reaction time: NR
Tsai et al. (2019)	M	Mild cognitive impairment	40 min × 3/week in 16 weeks	P2 and P3	Task switching paradigm	Amplitude: P2↔ P3↑	Accuracy:↑
						Latency: P2↔ P3↔	Reaction time:↑

**Notes.**

Shows results for all ERPs investigated with different cognitive paradigms using chronic aerobic exercise interventions. Arrows (↑) indicate increase in measure following exercise, arrows (↓) indicate decrease in measure following exercise and arrows ( ↔) indicate no difference.

HR Heart rate NR not registered CNV contingent negative variation RewP reward positivity ERN error-related negativity

## Discussion

The aim of the present study was to carry out a systematic review assessing the impact of aerobic exercise on ERPs related to cognitive performance. Heterogeneity across studies regarding methodology limited the possibility to draw definitive conclusions but the most consistent findings were that acute aerobic exercise was associated with higher amplitudes, and to a lesser extent shorter latencies, of ERPs. Moderate-intensity exercise was the most effective exercise intensity across studies in terms of affecting ERPs. For chronic exercise only a few studies were identified and results were less consistent. Lastly, in about half of the studies reporting an effect on ERPs, behavioral outcomes were also affected by the interventions.

Our findings are consistent with previous findings in another systematic review that evaluated the influence of physical activity or exercise on P3 in elderly participants. The authors concluded that physical activity and physical exercise positively influences changes in amplitude (Pedroso et al., 2017). Findings also suggested that P3 latency was less sensitive to physical activity compared to amplitude (Pedroso et al., 2017), which also aligns with the findings of the present systematic review.

P3 was the most frequently reported ERP in both acute and chronic exercise interventions in the identified studies. P3 represents the amount of attentional resources that is allocated to a specific task. Shorter latencies represent faster processing and higher amplitudes may be associated with attentional functioning (Polich & Heine, 1996). Our findings suggest that acute and to a lesser extent chronic exercise interventions seem to affect P3 amplitude. This aligns well with other studies that have found that exercise had a positive impact on attentional functioning and cognitive performance (Northey et al., 2018; Radel, Tempest & Brisswalter, 2018; Waters et al., 2020).

In the identified studies, other ERPs were also investigated such as the N1, N140 and N2 with the latter being the most frequently reported. Here, findings were more conflicting with studies reporting increases, decreases and no effects on amplitude and latency. These discrepancies may be due to difference in terms of intervention, control condition, paradigm used and study population, and further conclusions regarding these ERPs in relation to exercise are not possible.

We found that effects on ERPs seemed dependent on the exercise intensity, as most significant results for amplitudes were found in studies using moderate-intensity exercise. It has been proposed that P3 amplitude changes may be described by an inverted U-shaped curve relative to exercise intensity (Kamijo et al., 2004) and results from our study support this. It is uncertain what may mediate the U-shaped relationship indicating that low-intensity is “not enough” whereas high-intensity is “too much”. Interestingly, in a meta-analysis of patients with dementia, lower-frequency exercise interventions were more effective in improving cognition than higher-frequency interventions (Groot et al., 2016a) also hinting at the concept that not all doses of exercise are beneficial. In an observational study, a differential effect of exercise on cognitive functions was found, as physical activity was found to be positively associated with executive function and processing speed and negatively with memory (Frederiksen et al., 2015). Further studies are needed however, as results are conflicting (Hoffmann et al., 2016), and studies comparing different exercise intensities directly are few (Kamijo et al., 2007; Kao et al., 2017; Wang et al., 2016).

The use of ERPs in measuring cognitive performance post-exercise is practical and informative as the method enables assessment immediately before and after exercise. Further, temporal sensitivity is high, so ERP components can be tracked during cognitive paradigms. However, the spatial sensitivity is lacking and the different ERP components are difficult to locate (Woodman, 2010). Linking ERP findings with structural and functional MRI would give valuable information in this regard. However, studies are lacking in which MRI pre- and post-exercise has been carried out, especially in studies using acute exercise interventions. Future research should focus on concomitant use of MRI and ERPs in the investigation of cognitive responses, as the methods are complementary.

A number of aspects that possibly affect ERPs in relation to cognitive performance include age, exercise modality and exercise duration. We therefore divided the studies in acute and long-term aerobic exercise, although it can be theorized that smaller distinctions in duration could as well affect ERPs differently, *e.g.*, an exercise session under 20 min versus over 20 min. The majority of the studies included using acute exercise interventions had exercise duration between 9–40 min and no apparent difference was observed. Age related differences are also worth taking into account when interpreting ERP results, as studies have shown a latency increase and a P3 amplitude decrease in healthy senior individuals compared to younger individuals. Healthy seniors compared to individuals with dementia show further increase in latencies and decrease in amplitudes, which had led to the suggestion that P3 could be considered as a biological marker of cognitive impairment (Hedges et al., 2016; O’Mahony et al., 1996; Pedroso et al., 2012). Elderly individuals’ ERPs post-exercise could therefore be more susceptible to exercise than younger individuals (Hillman et al., 2002). Another ERP component that was frequently reported was N2, which is involved in inhibitory control. An increase in N2 amplitude is found to correlate with correctly inhibited no-go stimuli in both younger and older adults, whereas P3 amplitude in the same study showed an age-related decrease (Kardos, Kóbor & Molnár, 2020). This adds several variables to the issue, where it seems that some ERP components are age-dependent and some are not, but included studies in the systematic review investigating N2 were few and we refrain from concluding anything based on these results.

Several limitations were present in the studies included in the systematic review. There were concerns regarding risk of bias in all studies included. In study designs using aerobic exercise interventions it is almost impossible to blind participants, as aerobic exercise is not possible to mask and therefore performance bias is a risk. Furthermore, the studies were also difficult to compare, as ERPs were elicited through different sensory modalities by various cognitive tests that examined different aspects of cognitive performance. The investigators most often also examined different ERPs, such as P3 or N2, making direct comparisons between studies difficult.

The systematic review also has limitations. We chose to include studies on populations that were both healthy and with different diseases in our synthesis, which could have biased our results. Studies reporting on diseased participants were few and in general no discernable trends in the findings convincingly indicated a different response between healthy participants and diseased. We chose to focus on aerobic exercise interventions as these have shown more robust and consistent effects on cognition (Groot et al., 2016). However, other exercise types such as resistance training may have similar effects and therefore it cannot be ruled out that an effect on ERPs is also present for these types of exercise. We had very broad inclusion criteria in terms of paradigms and ERPs and having instead focused on one or two paradigms and ERPs would have perhaps left less uncertainty in terms of interpretation and may have enabled a meta-analysis. However, by including as many paradigms and ERPs as possible, we will enable researchers in having an overview of those used in exercise research. Thus we here present a wider review compared to the previous systematic review both in terms of population, intervention and ERPs examined (Pedroso et al., 2017).

In conclusion, we found that aerobic exercise, especially acute exercise, affected amplitudes and also to a lesser extent latencies of ERP components. Most studies focused on acute aerobic exercise in healthy participants and future research should focus more on (1) which role acute versus chronic exercise play in regards to ERP amplitudes and latencies, and (2) whether ERP amplitudes and latencies are dependent on exercise intensity. Future studies should focus on comparing these aspects of aerobic exercise directly.

##  Supplemental Information

10.7717/peerj.13604/supp-1 Supplemental Information 1 Exclusion reasons

10.7717/peerj.13604/supp-2 Supplemental Information 2 Search strategy

10.7717/peerj.13604/supp-3 Supplemental Information 3 PRISMA checklist

10.7717/peerj.13604/supp-4 Supplemental Information 4 Raw data extraction sheet

10.7717/peerj.13604/supp-5 Supplemental Information 5 Rationale for conducting this systematic review

10.7717/peerj.13604/supp-6 Supplemental Information 6 Risk of bias assessment

## Additional Information and Declarations

### Data Availability

The following information was supplied regarding data availability: The extraction sheet with all data on the studies included is available in the Supplementary File.

## References

[ref-1] Akatsuka K, Yamashiro K, Nakazawa S, Mitsuzono R, Maruyama A (2015). Acute aerobic exercise influences the inhibitory process in the go/no-go task in humans. Neuroscience Letters.

[ref-2] Aly M, Kojima H (2020). Acute moderate-intensity exercise generally enhances neural resources related to perceptual and cognitive processes: a randomized controlled ERP study. Mental Health and Physical Activity.

[ref-3] Bae S, Masaki H (2019). Effects of acute aerobic exercise on cognitive flexibility required during task-switching paradigm. Frontiers in Human Neuroscience.

[ref-4] Barha CK, Galea LA, Nagamatsu LS, Erickson KI, Liu-Ambrose T (2017). Personalising exercise recommendations for brain health: considerations and future directions. British Journal of Sports Medicine.

[ref-5] Brush CJ, Hajcak G, Bocchine AJ, Ude AA, Muniz KM, Foti D, Alderman BL (2022). A randomized trial of aerobic exercise for major depression: examining neural indicators of reward and cognitive control as predictors and treatment targets. Psychological Medicine.

[ref-6] Chacko SC, Quinzi F, Fano ADe, Bianco V, Mussini E, Berchicci M, Perri RL, Di Russo F (2020). A single bout of vigorous-intensity aerobic exercise affects reactive, but not proactive cognitive brain functions. International Journal of Psychophysiology.

[ref-7] Chang Y-K, Alderman BL, Chu C-H, Wang C-C, Song T-F (2017). Acute exercise has a general facilitative effect on cognitive function: a combined ERP temporal dynamics and BDNF study. Psychophysiology.

[ref-8] Chang Y-K, Pesce C, Chiang Y-T, Kuo C-Y, Fong D-Y (2015). Antecedent acute cycling exercise affects attention control: an ERP study using attention network test. Frontiers in Human Neuroscience.

[ref-9] Chen Y, Lu Y, Zhou C, Wang X (2020). The effects of aerobic exercise on working memory in methamphetamine-dependent patients: evidence from combined fNIRS and ERP. Psychology of Sport and Exercise.

[ref-10] Chu CH, Alderman BL, Wei GX, Chang YK (2015). Effects of acute aerobic exercise on motor response inhibition: an ERP study using the stop-signal task. Journal of Sport and Health Science.

[ref-11] Chu C-H, Kramer AF, Song T-F, Wu C-H, Hung T-M, Chang Y-K (2017). Acute exercise and neurocognitive development in preadolescents and young adults: an ERP study. Neural Plasticity.

[ref-12] Colcombe SJ, Erickson KI, Scalf PE, Kim JS, Prakash R, McAuley E, Elavsky S, Marquez DX, Hu L, Kramer AF (2006). Aerobic exercise training increases brain volume in aging humans. Journals of Gerontology - Series A Biological Sciences and Medical Sciences.

[ref-13] Dimitrova J, Hogan M, Khader P, O’Hora D, Kilmartin L, Walsh JC, Roche R, Anderson-Hanley CJ, Anderson-Hanley C (2017). Comparing the effects of an acute bout of physical exercise with an acute bout of interactive mental and physical exercise on electrophysiology and executive functioning in younger and older adults. Aging Clinical and Experimental Research.

[ref-14] Drapsin M, Radjo I, Klasnja A, Pasternak J, Krneta Z, Drid P (2012). Physical exercise and its influence on evoked cognitive potentials in the female subjects. Healthmed.

[ref-15] Duncan CC, Barry RJ, Connolly JF, Fischer C, Michie PT, Näätänen R, Polich J, Reinvang I, Van Petten C (2009). Event-related potentials in clinical research: guidelines for eliciting, recording, and quantifying mismatch negativity, P300, and N400. Clinical Neurophysiology.

[ref-16] Erickson KI, Hillman C, Stillman CM, Ballard RM, Bloodgood B, Conroy DE, Macko R, Marquez DX, Petruzzello SJ, Powell KE (2019). Physical activity, cognition, and brain outcomes: a review of the 2018 physical activity guidelines. Medicine Science in Sports Exercise.

[ref-17] Erickson KI, Voss MW, Prakash RS, Basak C, Szabo A, Chaddock L, Kim JS, Heo S, Alves H, White SM, Wojcicki TR, Mailey E, Vieira VJ, Martin SA, Pence BD, Woods JA, McAuley E, Kramer AF (2011). Exercise training increases size of hippocampus and improves memory. Proceedings of the National Academy of Sciences of the United States of America.

[ref-18] Frederiksen KS, Verdelho A, Madureira S, Bäzner H, O’Brien JT, Fazekas F, Scheltens P, Schmidt R, Wallin A, Wahlund LO, Erkinjunttii T, Poggesi A, Pantoni L, Inzitari D, Waldemar G (2015). Physical activity in the elderly is associated with improved executive function and processing speed: the ladis study. International Journal of Geriatric Psychiatry.

[ref-19] Gajewski PD, Falkenstein M (2018). ERP and behavioral effects of physical and cognitive training on working memory in aging: a randomized controlled study. Neural Plasticity.

[ref-20] Groot C, Hooghiemstra AM, Raijmakers PGHM, Van Berckel BNM, Scheltens P, Scherder EJA, Vander Flier WM, Ossenkoppele R (2016). The effect of physical activity on cognitive function in patients with dementia: a meta-analysis of randomized control trials. Ageing Research Reviews.

[ref-22] Hedges D, Janis R, Mickelson S, Keith C, Bennett D, Brown BL (2016). P300 amplitude in alzheimer’s disease. Clinical EEG and Neuroscience.

[ref-23] Heil M, Osman A, Wiegelmann J, Rolke B, Hennighausen E (2000). N200 in the Eriksen-task: inhibitory executive processes?. Journal of Psychophysiology.

[ref-24] Helfrich RF, Knight RT (2019). Cognitive neurophysiology: event-related potentials. Handbook of clinical neurology.

[ref-25] Hillman CH, Weiss EP, Hagberg JM, Hatfield BD (2002). The relationship of age and cardiovascular fitness to cognitive and motor processes. Psychophysiology.

[ref-26] Hoffmann K, Sobol NA, Frederiksen KS, Beyer N, Vogel A, Vestergaard K, Brændgaard H, Gottrup H, Lolk A, Wermuth L, Jacobsen S, Laugesen LP, Gergelyffy RG, Hogh P, Bjerregaard E, Andersen BB, Siersma V, Johannsen P, Cotman CW, Hasselbalch SG (2016). Moderate-to-high intensity physical exercise in patients with Alzheimer’s disease: a randomized controlled trial. Journal of Alzheimer’s Disease.

[ref-27] Hwang R-J, Chen H-J, Guo Z-X, Lee Y-S, Liu T-Y (2019). Effects of aerobic exercise on sad emotion regulation in young women: an electroencephalograph study. Cognitive Neurodynamics.

[ref-28] Jain P, Aprajita Jain P, Jain AK, Babbar R (2014). Influence of affective changes on behavioral and cognitive performances after acute bout of exhaustive exercise. Journal of Psychophysiology.

[ref-29] Kamijo K, Hayashi Y, Sakai T, Yahiro T, Tanaka K, Nishihira Y (2009). Acute effects of aerobic exercise on cognitive function in older adults. The Journals of Gerontology. Series B, Psychological Sciences and Social Sciences.

[ref-30] Kamijo K, Nishihira Y, Hatta A, Kaneda T, Wasaka T, Kida T, Kuroiwa K (2004). Differential influences of exercise intensity on information processing in the central nervous system. European Journal of Applied Physiology.

[ref-31] Kamijo K, Nishihira Y, Higashiura T, Kuroiwa K (2007). The interactive effect of exercise intensity and task difficulty on human cognitive processing. International Journal of Psychophysiology: Official Journal of the International Organization of Psychophysiology.

[ref-32] Kao SC, Drollette ES, Ritondale JP, Khan N, Hillman CH (2018). The acute effects of high-intensity interval training and moderate-intensity continuous exercise on declarative memory and inhibitory control. Psychology of Sport and Exercise.

[ref-33] Kao SC, Wang CH, Hillman CH (2020). Acute effects of aerobic exercise on response variability and neuroelectric indices during a serial n-back task. Brain and Cognition.

[ref-34] Kao S-C, Westfall DR, Soneson J, Gurd B, Hillman CH (2017). Comparison of the acute effects of high-intensity interval training and continuous aerobic walking on inhibitory control. Psychophysiology.

[ref-35] Kardos Z, Kóbor A, Molnár M (2020). Accurate response selection and inhibition in healthy aging: an event-related potential study. Psychology and Aging.

[ref-36] Krogh J, Rostrup E, Thomsen C, Elfving B, Videbech P, Nordentoft M (2014). The effect of exercise on hippocampal volume and neurotrophines in patients with major depression-a randomized clinical trial. Journal of Affective Disorders.

[ref-37] Lambourne K, Tomporowski P (2010). The effect of exercise-induced arousal on cognitive task performance: a meta-regression analysis. Brain Research.

[ref-38] Laurin D, Verreault R, Lindsay J, MacPherson K, Rockwood K (2001). Physical activity and risk of cognitive impairment and dementia in elderly persons. Archives of Neurology.

[ref-39] Ligeza TS, Maciejczyk M, Kałamała P, Szygula Z, Wyczesany M (2018). Moderate-intensity exercise boosts the N2 neural inhibition marker: a randomized and counterbalanced ERP study with precisely controlled exercise intensity. Biological Psychology.

[ref-40] Magnié MN, Bermon S, Martin F, Madany-Lounis M, Suisse G, Muhammad W, Dolisi C (2000). P300, N400, aerobic fitness, and maximal aerobic exercise. Psychophysiology.

[ref-41] Milankov M, Otto VukadinBarak, Sekulic AS, Milankov VM (2012). Event related potentials after acute bouts of exercise at different intensities in female non athletes and their relationship with sex hormones. Healthmed.

[ref-42] Nakamura Y, Nishimoto K, Akamatu M, Takahashi M, Maruyama A (1999). The effect of jogging on P300 event related potentials. Electromyography and Clinical Neurophysiology.

[ref-43] Northey JM, Cherbuin N, Pumpa KL, Smee DJ, Rattray B (2018). Exercise interventions for cognitive function in adults older than 50: a systematic review with meta-Analysis. British Journal of Sports Medicine.

[ref-44] Olson RL, Brush CJ, Ehmann PJ, Alderman BL (2017). A randomized trial of aerobic exercise on cognitive control in major depression. Clinical Neurophysiology : Official Journal of the International Federation of Clinical Neurophysiology.

[ref-45] O’Mahony D, Coffey J, Murphy J, O’Hare N, Hamilton D, Rowan M, Freyne P, Walsh JB, Coakley D (1996). Event-related potential prolongation in Alzheimer’s disease signifies frontal lobe impairment: evidence from SPECT imaging. Journals of Gerontology - Series A Biological Sciences and Medical Sciences.

[ref-46] Overath CH, Darabaneanu S, Evers MC, Gerber W-D, Graf M, Keller A, Niederberger U, Schäl H, Siniatchkin M, Weisser B (2014). Does an aerobic endurance programme have an influence on information processing in migraineurs?. The Journal of Headache and Pain.

[ref-47] Özkaya GY, Aydin H, Toraman FN, Kizilay F, Ozdemir O, Cetinkaya V (2005). Effect of strength and endurance training on cognition in older people. Journal of Sports Science & Medicine.

[ref-48] Pajonk FG, Wobrock T, Gruber O, Scherk H, Berner D, Kaizl I, Kierer A, Müller S, Oest M, Meyer T, Backens M, Schneider-Axmann T, Thornton AE, Honer WG, Falkai P (2010). Hippocampal plasticity in response to exercise in schizophrenia. Archives of General Psychiatry.

[ref-49] Pedroso RV, Fraga FJ, Corazza DI, Andreatto CAA, de MCoelhoFG, Costa JLR, Santos-Galduróz RF (2012). P300 latency and amplitude in Alzheimer’s disease: a systematic review. Brazilian Journal of Otorhinolaryngology.

[ref-50] Pedroso RV, Fraga FJ, Ayán C, Cancela Carral JM, Scarpari L, Santos-Galduróz RF (2017). Effects of physical activity on the P300 component in elderly people: a systematic review. Psychogeriatrics.

[ref-51] Pedroso RV, Cancela JM, Ayán C, Stein AM, Fuzaro G, Costa JLR, Fraga FJ, Santos-Galduróz RF (2018). Effects of physical exercise on the P300 of elderly with alzheimer’s disease. Journal of Physical Activity & Health.

[ref-52] Perini R, Bortoletto M, Capogrosso M, Fertonani A, Miniussi C (2016). Acute effects of aerobic exercise promote learning. Scientific Reports.

[ref-53] Polich J (2007). Subject, experience letter. Clinical Neurophysiology.

[ref-54] Polich J, Heine MRD (1996). P300 topography and modality effects from a single-stimulus paradigm. Psychophysiology.

[ref-55] Pontifex MB, Parks AC, Henning DA, Kamijo K (2015). Single bouts of exercise selectively sustain attentional processes. Psychophysiology.

[ref-56] Pope SK, Shue VM, Beck C (2003). Will a healthy lifestyle help prevent Alzheimer’s disease?. Annual Review of Public Health.

[ref-57] Radel R, Tempest GD, Brisswalter J (2018). The long and winding road: effects of exercise intensity and type upon sustained attention. Physiology and Behavior.

[ref-58] Ratey JJ, Loehr JE (2011). The positive impact of physical activity on cognition during adulthood: a review of underlying mechanisms, evidence and recommendations. Reviews in the Neurosciences.

[ref-59] Rietz E, Barker AR, Michelini G, Rommel AS, Vainieri I, Asherson P, Kuntsi J (2019). Beneficial effects of acute high-intensity exercise on electrophysiological indices of attention processes in young adult men. Behavioural Brain Research.

[ref-60] Scudder MR, Drollette ES, Pontifex MB, Hillman CH (2012). Neuroelectric indices of goal maintenance following a single bout of physical activity. Biological Psychology.

[ref-61] Shibasaki M, Namba M, Kamijo Y-I, Ito T, Kakigi R, Nakata H (2019). Effects of repetitive exercise and thermal stress on human cognitive processing. Physiological Reports.

[ref-62] Smith PJ, Blumenthal JA, Hoffman BM, Cooper H, Strauman TA, Welsh-Bohmer K, Browndyke JN, Sherwood A (2010). Aerobic exercise and neurocognitive performance: a meta-analytic review of randomized controlled trials. Psychosomatic Medicine.

[ref-63] Sokhadze EM, Casanova MF, Casanova E, Lamina E, Kelly DP, Khachidze I (2017). Event-related potentials (ERP) in cognitive neuroscience research and applications. NeuroRegulation.

[ref-64] Swatridge K, Regan K, Staines WR, Roy E, Middleton LE (2017). The acute effects of aerobic exercise on cognitive control among people with chronic stroke. Journal of Stroke & Cerebrovascular Diseases.

[ref-65] Takuro H, Nishihira Y, Soung-Ryol K (2009). Changes in cognitive function, response, preparation, and arousal level following moderate exercise. Advances in Exercise and Sports Physiology.

[ref-66] Themanson JR, Hillman CH (2006). Cardiorespiratory fitness and acute aerobic exercise effects on neuroelectric and behavioral measures of action monitoring. Neuroscience.

[ref-67] Tsai CL, Chen FC, Pan CY, Wang CH, Huang TH, Chen TC (2014). Impact of acute aerobic exercise and cardiorespiratory fitness on visuospatial attention performance and serum BDNF levels. Psychoneuroendocrinology.

[ref-68] Tsai CL, Ukropec J, Ukropcová B, Pai MC (2018). An acute bout of aerobic or strength exercise specifically modifies circulating exerkine levels and neurocognitive functions in elderly individuals with mild cognitive impairment. NeuroImage: Clinical.

[ref-69] Tsai C-L, Pai M-C, Ukropec J, Ukropcova B (2019). Distinctive effects of aerobic and resistance exercise modes on neuro-cognitive and biochemical changes in individuals with mild cognitive impairment. Current Alzheimer Research.

[ref-70] Tsai C-L, Pan C-Y, Chen F-C, Tseng Y-T (2017). Open- and closed-skill exercise interventions produce different neurocognitive effects on executive functions in the elderly: a 6-month randomized, controlled trial. Frontiers in Aging Neuroscience.

[ref-71] Tsai CL, Pan CY, Tseng YT, Chen FC, Chang YC, Wang TC (2021). Acute effects of high-intensity interval training and moderate-intensity continuous exercise on BDNF and irisin levels and neurocognitive performance in late middle-aged and older adults. Behavioural Brain Research.

[ref-72] Voss MW, Weng TB, Narayana-kumanan K, Cole RC, Wharff C, Reist L, Dubose L, Schmidt PG, Mills JA, Long JD, Magnotta VA, Pierce GL (2021). Connectivity.

[ref-73] Walsh JJ, Colino FL, Krigolson OE, Luehr S, Gurd BJ, Tschakovsky ME (2019). High-intensity interval exercise impairs neuroelectric indices of reinforcement-learning. Physiology & Behavior.

[ref-74] Wang D, Zhu T, Chen J, Lu Y, Zhou C, Chang Y-K (2020). Acute aerobic exercise ameliorates cravings and inhibitory control in heroin addicts: evidence from event-related potentials and frequency bands. Frontiers in Psychology.

[ref-75] Wang D, Zhu T, Zhou C, Chang Y-K (2017). Aerobic exercise training ameliorates craving and inhibitory control in methamphetamine dependencies: a randomized controlled trial and event-related potential study. Psychology of Sport and Exercise.

[ref-76] Wen HJ, Tsai CL (2020). Effects of acute aerobic exercise combined with resistance exercise on neurocognitive performance in obesewomen. Brain Sciences.

[ref-77] Winneke AH, Huebner L, Godde B, Voelcker-Rehage C (2019). Moderate cardiovascular exercise speeds up neural markers of stimulus evaluation during attentional control processes. Journal of Clinical Medicine.

[ref-78] Wollseiffen P, Schneider S, Martin LA, Kerhervé HA, Klein T, Solomon C (2016). The effect of 6 h of running on brain activity, mood, and cognitive performance. Experimental Brain Research.

[ref-79] Won J, Wu S, Ji H, Smith JC, Park J (2017). Executive function and the p300 after treadmill exercise and futsal in college soccer players. Sports.

[ref-80] Wu C-H, Karageorghis CI, Wang C-C, Chu C-H, Kao S-C, Hung T-M, Chang Y-K (2019). Effects of acute aerobic and resistance exercise on executive function: an ERP study. Journal of Science and Medicine in Sport.

[ref-81] Xie C, Alderman BL, Meng F, Ai J, Chang Y-K, Li A (2020). Acute high-intensity interval exercise improves inhibitory control among young adult males with obesity. Frontiers in Psychology.

[ref-82] Yagi Y, Coburn KL, Estes KM, Arruda JE (1999). Effects of aerobic exercise and gender on visual and auditory P300, reaction time, and accuracy. European Journal of Applied Physiology and Occupational Physiology.

[ref-83] Yagi Y, Shimono M, Estes K, Coburn K, Hashimoto I, Kakigi R (1998). Changes in information processing during exercise. Recent advances in human neurophysiology.

[ref-84] Wang D, Zhou C, Zhao M, Wu X, Chang Y-K (2016). Dose–response relationships between exercise intensity, cravings, and inhibitory control in methamphetamine dependence: an ERPs study. Drug and Alcohol Dependence.

[ref-85] Waters A, Zou L, Jung M, Yu Q, Lin J, Liu S, Loprinzi PD (2020). Acute exercise and sustained attention on memory function. American Journal of Health Behavior.

[ref-86] Woodman GF (2010). A brief introduction to the use of event-related potentials in studies of perception and attention. Attention, Perception Psychophysics.

[ref-87] Zhao Q, Wang X, Lu Y, Zhao Q, Zhou C (2020). Effects of chronic exercise on temporal discounting among persons with methamphetamine use disorder. Mental Health and Physical Activity.

[ref-88] Zhou F, Qin C (2019). Acute moderate-intensity exercise generally enhances attentional resources related to perceptual processing. Frontiers in Psychology.

