# Supplementary material for: Effects of aerobic exercise on event-related potentials related to cognitive performance: a systematic review
Source: PeerJ. 2022 Jul 11;10:e13604. doi: 10.7717/peerj.13604 (PMC9281596; doi:10.7717/peerj.13604)
Supplement: Supplemental Information 1 [file peerj-10-13604-s001.docx]

**Supplementary**

Exclusion reasons

| **First author** | **Study** | **Main reason for exclusion** |
| --- | --- | --- |
| Chang, YK | Acute exercise has a general facilitative effect on cognitive function- A combined ERP temporal dynamics and BDNF study | Wrong outcome measure |
| Brush, CJ | Aerobic exercise enhances positive emotional reactivity in individuals with depressive symptoms- Evidence from neural responses to reward and emotional content | Wrong outcome measure |
| Chang, YK | Antecedent acute cycling exercise affects attention control- an ERP study using attention network test | Wrong outcome measure |
| Kamijo, K | Differential influences of exercise intensity on information processing in the central nervous system | Wrong outcome measure |
| Dustman, RE | EEG and event-related potentials in normal aging | Wrong study design |
| Kamijo, K | EFFECTS OF EXERCISE INTENSITY AND PHYSICAL ACTIVITY LEVELS ON THE BRAIN AND COGNITION | Wrong language |
| Özmerdivenli, R | Effects of exercise on visual evoked potentials | Wrong intervention |
| Tsai, CL | Executive function and endocrinological responses to acute resistance exercise | Wrong intervention |
| Kota, S | Feasibility of using event-related potentials as a sideline measure of neurocognitive dysfunction during sporting events | Wrong study design |
| Maruo, Y | Long-Distance Runners and Sprinters Show Different Performance Monitoring - An Event-Related Potential Study | Wrong study design |
| Polich, T | P300 and long-term physical exercise | Wrong study design |
| O’Leary, KC | The effects of single bouts of aerobic exercise, exergaming, and videogame play on cognitive control | A thesis |
| Thom, NJ | Acute Exercise Prevents Angry Mood Induction but Does Not Change Angry Emotions | Wrong outcome measure |
| Wang, CH | Aerobic exercise modulates transfer and brain signal complexity following cognitive training. | Wrong outcome measure |
| Li, Y | Characteristics of brainstem auditory evoked potentials of students studying folk dance | Wrong study design |
| Zwierko, T | Does athletic training in volleyball modulate the components of visual evoked potentials A preliminary investigation | Wrong study population |
| Wang, D | Dose-response relationships between exercise intensity, cravings, and inhibitory control in methamphetamine dependence- An ERPs study. | Wrong outcome measure |
| Zhu, Y | Effects of a specially designed aerobic dance routine on mild cognitive impairment | Wrong outcome measure |
| Lu, Y | Effects of exercise programs on neuroelectric dynamics in drug addiction | Wrong outcome measure |
| Schmidt-Kassow, M | Exercising during learning improves vocabulary acquisition- behavioral and ERP evidence | Wrong intervention |
| Wu, S | Exergaming Improves Executive Functions in Patients With Metabolic Syndrome- Randomized Controlled Trial | Wrong outcome measure |
| Yamazaki, Y | Modulation of inhibitory function in the primary somatosensory cortex and temporal discrimination threshold induced by acute aerobic exercise | Wrong outcome measure |
| Bullock, T | Multiple stages of information processing are modulated during acute bouts of exercise | Wrong outcome measure |
| Vogt, T | Neuroelectric adaptations to cognitive processing in virtual environments- an exercise-related approach | Wrong outcome measure |
| Olson, RL | Neurophysiological and behavioral correlates of cognitive control during low and moderate intensity exercise | Wrong outcome measure |
| Yamashiro, K | Skill-Specific Changes in Somatosensory Nogo Potentials in Baseball Players. | Wrong study design |
| Yamashiro, K | Skill-specific changes in somatosensory-evoked potentials and reaction times in baseball players | Wrong outcome measure |
| Tsai, CL | The effects of different exercise types on visuospatial attention in the elderly | Wrong study design |
| Zoshk, MRY | The Measurement and Processing of EEG Signals to Evaluate Fatigue | Wrong intervention |
| Ligeza, TZ | Acute aerobic exercise enhances pleasant compared to unpleasant visual scene processing | Wrong outcome measure |
| Amjad, I | Acute effects of aerobic exercise on somatosensory-evoked potentials in patients with mild cognitive impairment | Wrong outcome measure |
| Jones, MD | Aerobic Exercise Reduces Pressure More Than Heat Pain Sensitivity in Healthy Adults | Wrong outcome measure |
| Spitzer, MWH | Being physically active versus watching physical activity - Effects on inhibitory control | Wrong population |
| Cetin, E | Effect of vitamin E supplementation with exercise on cognitive functions and total antioxidant capacity in older people. | Wrong intervention |
| Nakata, H | Effects of aerobic exercise under different thermal conditions on human somatosensory processing. | Wrong outcome measure |
| Shete, AN | Effects of Exercise on Visual Evoked Potentials | Wrong outcome measure |
| Gowsi, K | Effects of moderate and high-intensity exercise on P300 latency and reaction time in athletes and nonathletes - An interim analysis | Wrong outcome measure |
| Barak, O | Event-related potentials following exercise bouts of different intensity | Wrong language |
| Tsai, CL | Exercise-mode-related changes in task-switching performance in the elderly | Wrong study design |
| Jones, MD | Exploring The Mechanisms Of Exercise-induced Hypoalgesia Using Somatosensory Evoked Potentials | Wrong outcome measure |
| Fearnbach, SN | Reduced neural response to food cues following exercise is accompanied by decreased energy intake in obese adolescents | Wrong population |
| Marinella, C | Relationship of high blood lactate levels with latency of visual-evoked potentials. | Wrong outcome measure |
| Ludyga, S | The effects of a school-based exercise program on neurophysiological indices of working memory operations in adolescents | Wrong population |
| Brown, KE | The influence of an acute bout of moderate-intensity cycling exercise on sensorimotor integration | Wrong outcome measure |
| Laine, A | Acute effects of 1,1,1-trichloroethane inhalation on the human central nervous system. | Wrong intervention |
| Özbay, EA | AN ERP STUDY ON EFFECTS OF COMPLEX MOTOR MOVEMENT TRAINING ON FOOTBALL PLAYERS' SUSTAINED ATTENTION PERFORMANCE | Wrong language |
| Chang, YK | Antecedent acute cycling exercise affects attention control- An ERP study using attention network test | Wrong outcome measure |
| Chang, YK | Effect of acute exercise and cardiovascular fitness on cognitive function- An event-related cortical desynchronization study | Wrong outcome measure |
| Bradford, JC | Effect of locomotor demands on cognitive processing | Wrong outcome measure |
| Tokuda, H | Effects of combining exercise with long-chain polyunsaturated fatty acid supplementation on cognitive function in the elderly- a randomised controlled trial | Wrong intervention |
| Dodwell, G | Electroencephalographic evidence for improved visual working memory performance during standing and exercise. | Wrong outcome measure |
| Kubova, Z | Influence of physiological changes of glycemia on VEPs and visual ERPs | Wrong intervention |
| Hanlon, B | Neural response to pictures of food after exercise in normal-weight and obese women | Wrong outcome measure |
| Hwang, RJ | The effect of aerobic exercise on the sad emotional processing in menopausal women- An electroencephalography (eeg) study | Wrong outcome measure |
| Zwierko, T | The effect of progressively increased physical efforts on visual evoked potentials in volleyball players and non-athletes | Wrong outcome measure |
| Thom, NJ | The Effects of an Acute Bout of Moderate Intensity Exercise on Anger and EEG Responses During Elicitation of Angry Emotions | It is a dissertation |
| Zhao, X | Working Memory Updating Function Training Influenced Brain Activity | Wrong intervention |
| Popovich, C | Acute aerobic exercise enhances attentional modulation of somatosensory event-related potentials during a tactile discrimination task | Wrong outcome measure |
| Seppalainen, AM | Changes induced by short-term xylene exposure in human evoked potentials | Wrong intervention |
| Akiyama, S | Event-related potentials (ERPs) and long-term physical exercise | Wrong language |
| Wildberger, H | Fluctuations of visual evoked potential amplitudes and of contrast sensitivity in Uhthoff's symptom | Wrong outcome measure |
| O’leary, TJ | High-intensity exhaustive exercise reduces long-interval intracortical inhibition | Wrong outcome measure |
| Pontifex, MB | Neuroelectric and behavioral indices of interference control during acute cycling | Wrong outcome measure |
| Scanlon, JEM | Taking off the training wheels- Measuring auditory P3 during outdoor cycling using an active wet EEG system | Wrong outcome measure |
| Attias, J | THE EFFECT OF MODERATE HEAT-STRESS ON AUDITORY BRAIN-STEM EVOKED-RESPONSE IN MAN | Wrong outcome measure |
| Magnie, MN | Visual and brainstem auditory evoked potentials and maximal aerobic exercise- Does the influence of exercise persist after body temperature recovery | Wrong outcome measure |
| Qingnian, H | The effects of long-term Qi gong exercise on brain function as manifested by computer analysis | Wrong study design |
| Gliner, JA | Visual evoked potentials and signal detection following a marathon race | Wrong outcome measure |
| Zhao, JG | Changes in the latencies of visual-evoked potentials in people undergoing tennis training – Dynamic comparison before and after 8 weeks training | Wrong outcome measure |
| Ozdemir, O | The effect of six months walking exercise and vitamin E supplementation on event-related potentials in older people | Conference abstract |
| Jain, P | Maximal exercise modulates event related potentials and reaction time in untrained medical students | Conference abstract |
| Meadows, C | Comparison of high-intensity interval training exercise modalities on executive funtion and temporal dynamics of inhibitory control and event-related potentials | Conference abstract |
| Frances, M | DOES EXERCISE ENHANCE COGNITIVE PROCESSING IN YOUNG AND MIDDLE-AGED ADULTS? | Poster abstract |
| Sage, M | Can an acute bout of aerobic exercise after stroke influence cortical somatosensory excitability? | Conference abstract |
| Krouse, R | Warm-up period in physical exercise in relation to brain potential | Wrong study design |
| Kaylie, CA | The effects of time of day and exercise on event-related potential indices of response inhibition to high-and low-calorie foods | Meeting abstract |
| Barthel, T | The effect of different levels of exercises on an ergometer on EEG and readyness potential (RP) | Wrong language |
| Smagin, NV | Study of the electrophysiological parameters of running with various intensities along a movable support | Wrong language |
| Shields, M | The effect of exercise on the late-positive potential evoked during affective picture viewing | Poster abstract |
| Wang, QR | Effect of early exercise therapy on the recovery of motor function in patients with cerebral infarction and the changes of somatosensory evoked potential | Wrong language |
| Itagi, ABH | Physical exhaustion induced variations in event-related potentials and cognitive task performance in young adults | Wrong outcome measure |
| Wang, D | Acute exercise ameliorates craving and inhibitory deficits in methamphetamine- An ERP study | Wrong outcome measure |
