# Supplementary material for: Effects of aerobic exercise on event-related potentials related to cognitive performance: a systematic review
Source: PeerJ. 2022 Jul 11;10:e13604. doi: 10.7717/peerj.13604 (PMC9281596; doi:10.7717/peerj.13604)
Supplement: Supplemental Information 2 [file peerj-10-13604-s002.docx]

**Search strategy**

Research question: how does aerobic exercise affect evoked potentials obtained by EEG/MEG?

**PubMed**

**Concept 1: Exercise**

**MeSH:** "Exercise"[Mesh]

**Concept 2: EEG**

**MeSH:** "Electroencephalography"[Mesh]

**Keywords:** EEG

**Concept 3: MEG**

**MeSH:** "Magnetoencephalography"[Mesh]

**Keywords:** MEG

**Concept 4: Evoked potentials**

**MeSH:** "Evoked Potentials"[Mesh]

**Keywords:** event-related potential*

Search:


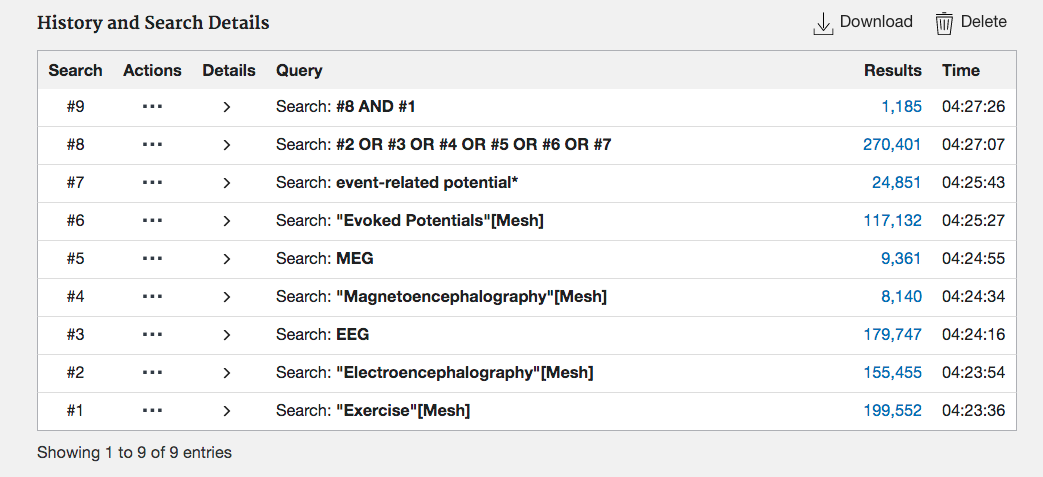


Search made 05/11/2020

**Web of Science**

**Concept 1: Exercise**

**Keywords:** TS=(exercise)

**Concept 2: EEG**

**Keywords:** TS=(electroencephalography) OR TS=(EEG)

**Concept 3: MEG**

**Keywords:** TS=(magnetoencephalography) OR TS=(MEG)

**Concept 4: Evoked potentials**

**Keywords:** TS=(evoked potential*) OR TS=(event-related potential*)

Search:


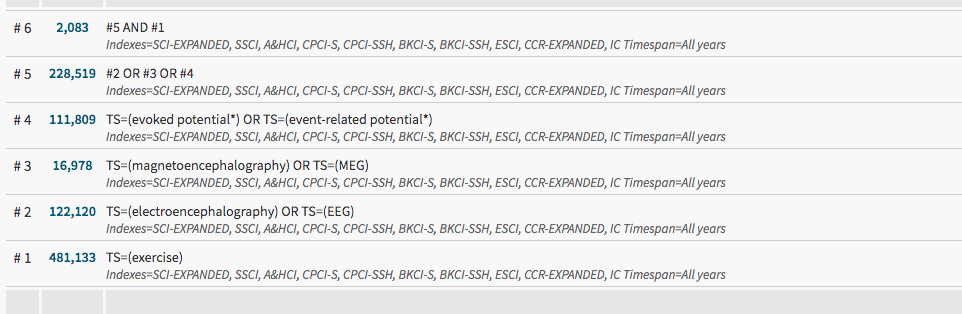


Search made 05/11/2020

**Cochrane library**

**Concept 1: Exercise**

**MeSH:** MeSH descriptor: [Exercise] explode all trees

**Concept 2: EEG**

**MeSH:** MeSH descriptor: [Electroencephalography] explode all trees

**Keywords:** EEG

**Concept 3: MEG**

**MeSH:** MeSH descriptor: [Magnetoencephalography] explode all trees

**Keywords:** MEG

**Concept 4: Evoked potentials**

**MeSH:** MeSH descriptor: [Evoked Potentials] explode all trees

**Keywords:** even-related potential

Search


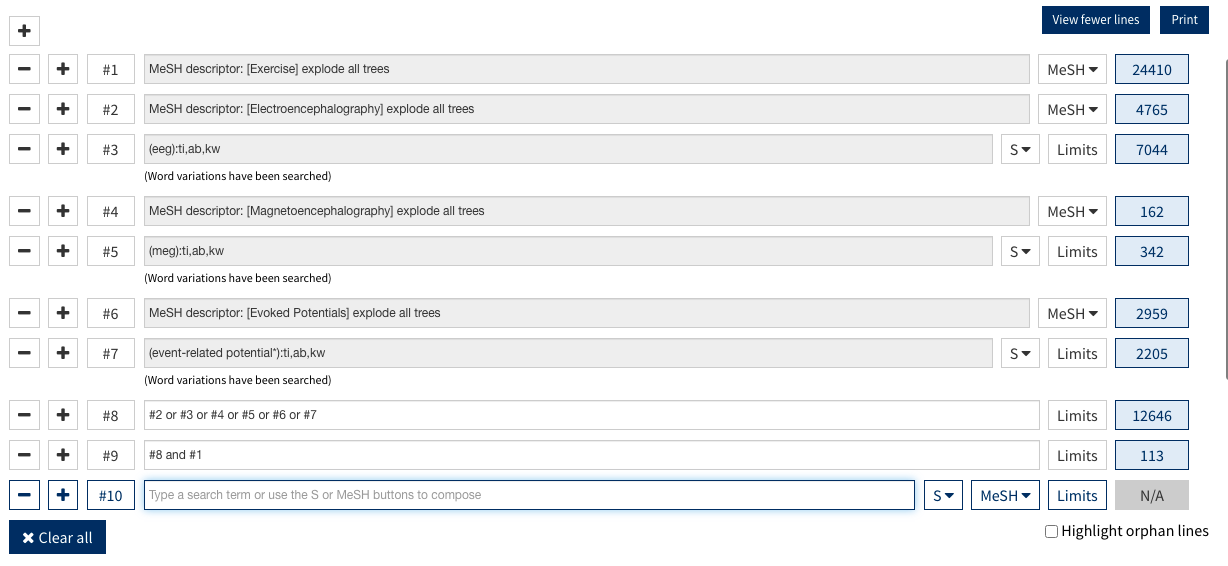


Search made 06/11/2020

**Embase**

**Concept 1: Exercise**

**Subject heading:** exp exercise/

**Keywords:** exercise.mp.

**Concept 2: EEG**

**Subject heading:** exp electroencephalography/

**Keywords:** electroencephalography.mp. OR EEG.mp.

**Concept 3: MEG**

**Subject heading:** exp magnetoencephalography/

**Keywords:** magnetoencephalography.mp OR MEG.mp

**Concept 4: Evoked potentials**

**Subject heading:** exp evoked response/ OR exp event-related potential/

**Keywords:** evoked potential.mp. OR event-related potential.mp.

Search:

**
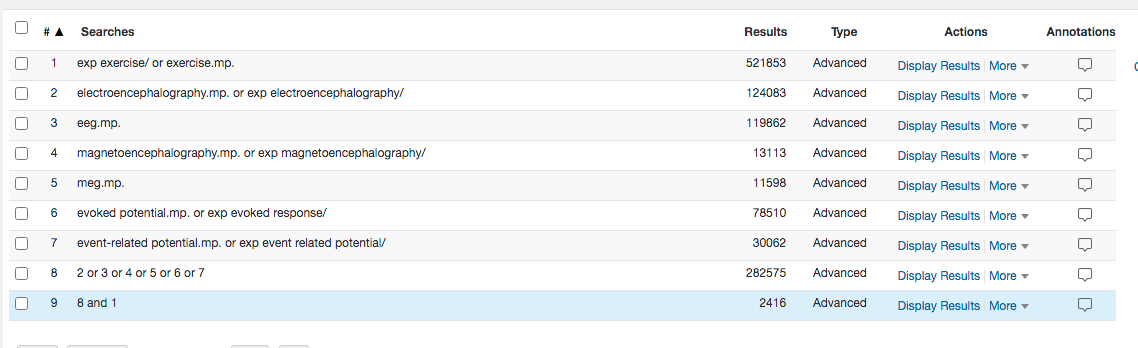
**

Search made 06/11/2020
