# Supplementary material for: Effects of aerobic exercise on event-related potentials related to cognitive performance: a systematic review
Source: PeerJ. 2022 Jul 11;10:e13604. doi: 10.7717/peerj.13604 (PMC9281596; doi:10.7717/peerj.13604)
Supplement: Supplemental Information 5 [file peerj-10-13604-s005.docx]

Effects of exercise on event-related potentials: a systematic review

**Rationale and contribution**

The rationale for conducting this systematic review was to investigate the effects of aerobic exercise on cognition measured as event-related potentials (ERPs). Studies have shown that physical exercise improves brain functioning, both brain health and cognition. Although the beneficial effects of exercise on brain functioning are mediated through many different pathways, we have focused on ERPs as it is an informative dynamic method to investigate and monitor information processing in the brain (Sokhadze et al., 2017). We divided the studies in two groups; studies that had chronic exercise and studies that had acute exercise as interventions. It is theorized that the effects of acute exercise are shorter lasting and that acute exercise possibly affects cognition in different ways than chronic exercise does. As an example, one study has shown that plasma brain-derived neurotrophic factor (BDNF), which mediates some of the beneficial effects of exercise (Etnier et al., 2016), was increased in response to acute exercise but that the effects of long-term aerobic exercise were less clear (Huang et al., 2014; Tsai et al., 2021). We also subdivided the studies in subgroups according to their exercise intensities, as studies have shown that aerobic low-, moderate- and high intensity exercise affect the brain differently and an inverted U-shaped relationship between exercise intensity and P3 amplitude has been proposed (Kamijo et al., 2004). Moderate-intensity exercise has also been shown to be most effective in improving inhibitory control and electrical measures in methamphetamine-depended individuals post-exercise (Wang et al., 2016).

Exercise is a cost-beneficial way to improve brain functioning. We believe that there is plenty of evidence on the beneficial effects of exercise, but that the issue should be investigated more thoroughly to be able to conclude aerobic exercise to be an efficient way to promote brain health and cognition.

The systematic review contributes with added knowledge on how aerobic exercise affects the brain. A previously published systematic review, comparable to ours, included studies where P300 was investigated post-exercise in elderly. They found that “physical exercise, especially those involving aerobic or resistance training, seems to have marked beneficial effects on P300 in the elderly. Evidence shows that physical exercise positively influences cortical activities related to cognitive functions, as indicated by P300, in elderly people” (Pedroso et al., 2017). These findings support our results, but we investigated in broader populations and concluded that the effects of exercise on P300 also extend to a younger population. We also included studies that examined other ERP components, which led to the finding that the effects extended to more ERP components than P300.

**References**

Etnier, B. J. L., Wideman, L., Labban, J. D., Piepmeier, A. T., Daniel, M., Dvorak, K. K., Becofsky, K., Jl, E., Wideman, L., Jd, L., Piepmeier, A., Dm, P., Dvorak, K., & Becofsky, K. (2016). *Journal of Sports and Exercise Psychology : 38(4): 331-340.* *2010*.

Huang, T., Larsen, K. T., Ried-Larsen, M., Møller, N. C., & Andersen, L. B. (2014). The effects of physical activity and exercise on brain-derived neurotrophic factor in healthy humans: A review. *Scandinavian Journal of Medicine & Science in Sports*, *24*(1), 1–10. https://doi.org/10.1111/sms.12069

Kamijo, K., Nishihira, Y., Hatta, A., Kaneda, T., Kida, T., Higashiura, T., & Kuroiwa, K. (2004). Changes in arousal level by differential exercise intensity. *Clinical Neurophysiology : Official Journal of the International Federation of Clinical Neurophysiology*, *115*(12), 2693–2698. https://doi.org/10.1016/j.clinph.2004.06.016

Pedroso, R. V., Fraga, F. J., Ayán, C., Cancela Carral, J. M., Scarpari, L., & Santos-Galduróz, R. F. (2017). Effects of physical activity on the P300 component in elderly people: a systematic review. *Psychogeriatrics*, *17*(6), 479–487. https://doi.org/10.1111/psyg.12242

Sokhadze, E. M., Casanova, M. F., Casanova, E., Lamina, E., Kelly, D. P., & Khachidze, I. (2017). Event-related potentials (ERP) in cognitive neuroscience research and applications. *NeuroRegulation*, *4*(1), 14–27. https://doi.org/10.15540/nr.4.1.14

Tsai, C. L., Pan, C. Y., Tseng, Y. T., Chen, F. C., Chang, Y. C., & Wang, T. C. (2021). Acute effects of high-intensity interval training and moderate-intensity continuous exercise on BDNF and irisin levels and neurocognitive performance in late middle-aged and older adults. *Behavioural Brain Research*, *413*(1), 113472. https://doi.org/10.1016/j.bbr.2021.113472

Wang, D., Zhou, C., Zhao, M., Wu, X., & Chang, Y.-K. (2016). Dose-response relationships between exercise intensity, cravings, and inhibitory control in methamphetamine dependence: An ERPs study. *Drug and Alcohol Dependence*, *161*, 331–339. https://doi.org/10.1016/j.drugalcdep.2016.02.023
