# Supplementary material for: Effects of aerobic exercise on event-related potentials related to cognitive performance: a systematic review
Source: PeerJ. 2022 Jul 11;10:e13604. doi: 10.7717/peerj.13604 (PMC9281596; doi:10.7717/peerj.13604)
Supplement: Supplemental Information 6 [file peerj-10-13604-s006.pdf]

Risk of bias

	Sequence generation	Allocation concealment	Blinding of participants, personnel and outcome assessors	Incomplete outcome data	Selective outcome reporting	Other sources of bias	Overall judgment
Xie et al. 2020							
Wang et al. 2017							
Tsai et al. 2013							
Yagi et al. 1999							
Pedroso et al. 2018							
Chang et al. 2016							
Walsh et al. 2019							
Scudder et al. 2012							
Tsai et al. 2017							
Swatridge et al. 2017							
Olson et al. 2017							
Chacko et al. 2020							
Akatsuka et al. 2015							
Kamijo et al. 2009							
Kao et al. 2020							
Aly et al. 2020							
Tsai et al. 2018							
Wollseifen et al. 2016							
Overath et al. 2014							

Chang et al. 2015							
Wen et al. 2020							
Chu et al. 2015							
Shibasaki et al. 2019							
Milankov et al. 2012							
Ligeza et al. 2018							
Pontifex et al. 2016							
Chen et al. 2020							
Kamijo et al. 2007							
Wang et al. 2020							
Chu et al. 2017							
Zhou et al. 2019							
Rietz et al. 2018							
Thermanson et al. 2006							
Dimitrova et al. 2016							
Kao et al. 2017							
Zhao et al. 2020							
Won et al. 2017							
Özkaya et al. 2005							
Brush et al. 2020							
Bae et al. 2019							
Hwang et al. 2018							
Magnie et al. 2000							

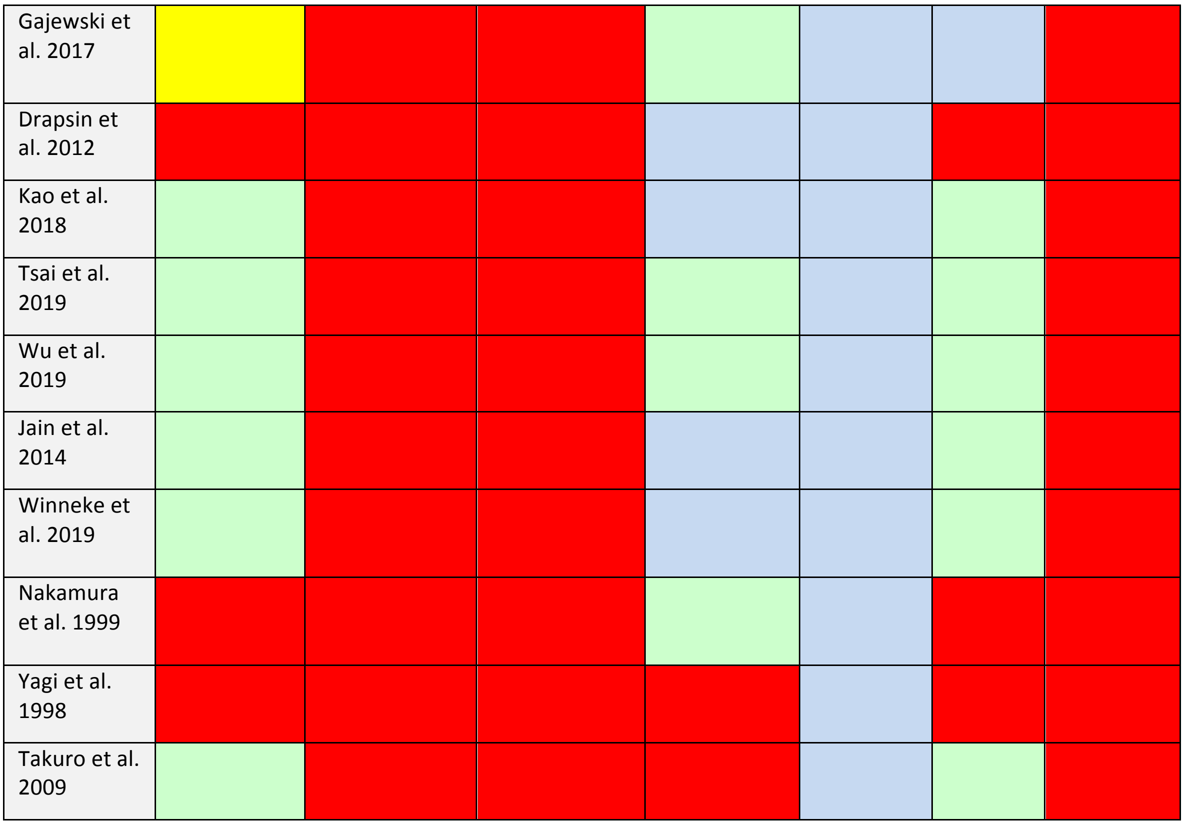

Green: low risk
Yellow: unclear
Red: high risk
Blue: no information
